# Structural Insights into the Receptor-Binding Domain of Bat Coronavirus HKU5-CoV-2: Implications for Zoonotic Transmission via ACE2

**DOI:** 10.3390/ani16020237

**Published:** 2026-01-13

**Authors:** Manal A. Babaker, Nariman Sindi, Othman Yahya Alyahyawy, Ehssan Moglad, Mohieldin Elsayid, Thamir M. Eid, Mohamed Eltaib Elmobark, Hisham N. Altayb

**Affiliations:** 1 Department of Chemistry, Faculty of Science, Majmaah University, Al Majmaah 11952, Saudi Arabia; m.babaker@mu.edu.sa; 2 Department of Medical Laboratory Sciences, Faculty of Applied Medical Sciences, King Abdulaziz University, Jeddah 21589, Saudi Arabia; nsindi@kau.edu.sa; 3 Department of Medical Laboratory Technology (MLT), Faculty of Applied Medical Sciences, King Abdulaziz University, Rabigh 80200, Saudi Arabia; oalyahyawy@kau.edu.sa; 4 Department of Pharmaceutics, College of Pharmacy, Prince Sattam bin Abdulaziz University, P.O. Box 173, Alkharj 11942, Saudi Arabia; ehssanhassn@gmail.com; 5 Clinical Laboratory Sciences-Jeddah, College of Applied Medical Sciences, King Saud bin Abdulaziz University for Health Sciences, King Abdulaziz Medical City, Jeddah 22384, Saudi Arabia; elsayidm@ksau-hs.edu.sa; 6 King Abdullah International Medical Research Center, King Abdulaziz Medical City, Jeddah 22384, Saudi Arabia; 7 Department of Biochemistry, Faculty of Science, King Abdulaziz University, Jeddah 21589, Saudi Arabia; tmeid@kau.edu.sa; 8 Experimental Biochemistry Unit, King Fahad Medical Research Center, King Abdulaziz University, Jeddah 21589, Saudi Arabia; 9 Department of Pharmaceutics, College of Pharmacy, Jazan University, Jazan 45142, Saudi Arabia; mohamedeltaib.me@gmail.com

**Keywords:** HKU5 coronavirus, zoonotic transmission, ACE2 receptor binding, molecular dynamics simulation, peptide inhibitor design

## Abstract

The zoonotic potential of bat coronaviruses, especially HKU5, is a significant issue because of their capacity to utilize human angiotensin-converting enzyme 2 (ACE2) as a receptor for cellular entry. This study offers structural insights into the binding kinetics of HKU5 (Bat Merbecovirus HKU5) receptor-binding domain (RBD) spike protein with human ACE2 through a multiscale computational method. This study employed structural modeling, 300-nanosecond (ns) molecular dynamics (MD) simulations, alanine-scanning mutagenesis, and computational peptide design to investigate ACE2 recognition by the HKU5 RBD and its interactions with peptides. The root mean square deviation (RMSD) investigation of HKU5–ACE2 complexes indicated that HKU5 exhibited greater flexibility than SARS-CoV-2, with RMSD values reaching a maximum of 1.2 nm. Free energy analysis, Molecular Mechanics/Generalized Born Surface Area (MM/GBSA), indicated a more robust binding affinity of HKU5 to ACE2 (ΔG_Total_ = −21.61 kcal/mol) in contrast to SARS-CoV-2 (ΔG_Total_ = −5.82 kcal/mol), implying that HKU5 binding with ACE2 had higher efficiency. Additionally, a peptide was designed from the ACE2 interface, resulting in the development of 380 single-site mutants by mutational alterations. The four most promising mutant peptides were selected for 300-nanosecond (ns) MD simulations, subsequently undergoing quantum chemical calculations (DFT) to evaluate their electronic characteristics. MM/GBSA of −37.83 kcal/mol indicated that mutant-1 exhibits the most favorable binding with HKU5, hence potentially inhibiting ACE2 interaction. Mutant-1 formed hydrogen bonds involving Glu74, Ser202, Ser204, and Asn152 residues of HKU5. Finally, QM/MM calculations on the peptide–HKU5 complexes showed the most favorable ΔE_interaction of −170.47 (Hartree) for mutant-1 peptide. These findings offer a thorough comprehension of receptor-binding dynamics and are crucial for evaluating the zoonotic risk associated with HKU5-CoV and guiding the design of receptor-targeted antiviral treatments.

## Simple Summary

Bats are recognized as carriers of many coronaviruses, some of which are capable of infecting people. The virus HKU5 (Bat Merbecovirus HKU5) may possess the capability to attach to the human cell receptor angiotensin-converting enzyme 2 (ACE2), which is also utilized by viruses such as SARS-CoV and SARS-CoV-2 for cellular entry. This study employed computational modeling and simulations to elucidate the attachment of the HKU5 virus to ACE2 and the strength of its binding. We also engineered small protein-like molecules, known as peptides, to evaluate their capacity to inhibit this interaction. Our findings indicate that HKU5 exhibits a stronger affinity for ACE2 compared to SARS-CoV-2, implying a greater potential for human cell infection. Among the evaluated peptides, a specific mutant variant demonstrated notably robust and sustained affinity for HKU5, indicating its potential to inhibit the virus’s attachment to ACE2. These findings enhance our comprehension of the infection risk associated with HKU5 and establish a basis for the development of novel antiviral therapies to prevent potential cross-species transmission.

## 1. Introduction

Emerging coronaviruses originating from bat reservoirs persist in threatening human health; the global impacts of SARS-CoV, MERS-CoV, and SARS-CoV-2 (Severe Acute/Middle East Respiratory Syndrome Coronavirus) serve as notable instances. The ability of viral spike proteins to attach to host entry receptors, particularly angiotensin-converting enzyme 2 (ACE2), is a crucial determinant in interspecies viral transmission [1,2]. While SARS-related coronaviruses have been well examined for ACE2-mediated entry, recent studies indicate that certain merbecoviruses, like HKU5-CoV, which infect bats, can also utilize this mechanism [3,4].

Research indicates that ACE2 functions as a receptor for HKU5 coronaviruses in *Pipistrellus abramus* bats, suggesting the potential for zoonotic transmission to humans or other intermediary species [5]. Further investigation into the HKU5-CoV lineage 2 has demonstrated that these viruses can infiltrate human cells via ACE2 [6,7]. This underscores the peril of bat reservoirs harboring latent infectious viruses. Close relatives of MERS-CoV exhibit convergent evolution in their receptor-binding domains, facilitating functional interactions with ACE2 orthologs across other taxa, as revealed by comparative studies of bat-derived merbecoviruses [8].

Peptide-based inhibitors have attracted significant interest as antiviral medicines owing to their excellent selectivity, adaptability, and compatibility with protein–protein interaction (PPI) interfaces. Numerous studies have effectively created peptide or mini-protein inhibitors aimed at the interactions between SARS-CoV-2 RBD and ACE2, exhibiting strong neutralization and experimental viability (e.g., LCB1 mini-protein, ACE2-derived helical peptides) [9,10,11]. Nonetheless, no peptide inhibitors have been formulated for HKU5 or other merbecoviruses, resulting in a significant therapeutic deficiency. Computational peptide design provides a systematic method to identify ACE2-derived inhibitory peptides that can compete with HKU5 for ACE2 binding, thus reducing the possibility of zoonotic spillover.

Structurally, HKU5 occupies an evolutionary position that is unique from both SARS-CoV and SARS-CoV-2. Although SARS-CoV and SARS-CoV-2 exhibit approximately 76% sequence commonality in their receptor-binding domains (RBDs), the HKU5 RBD demonstrates only about 55–60% identity with either virus, indicating significant divergence in conserved motifs and loop regions essential for ACE2 binding. Recent structural investigations indicate that the HKU5 RBD attains ACE2 recognition via a convergently evolved binding interface, despite the absence of several classical ACE2-contact residues found in SARS-CoV and SARS-CoV-2 [12]. Nonetheless, despite the recent availability of Cryo-EM structures of HKU5 coupled to ACE2 (e.g., PDB 9JJ6), the structural repertoire of the HKU5 spike remains incomplete, with absent loops and unresolved regions critical for precise molecular simulations. This structural incompleteness requires computational modeling to reconstruct absent residues and provide stable conformations appropriate for subsequent analyses.

Considering the accumulating evidence that HKU5-CoV may interact with human ACE2, it is imperative to elucidate its binding mechanism, pinpoint critical residues involved in receptor recognition, and evaluate its potential for zoonotic transmission. Comparative structural analysis with SARS-CoV-2, the most well-characterized ACE2-binding coronavirus, offers an essential framework for understanding HKU5’s host-adaptive characteristics. SARS-CoV-2 has specific molecular determinants of ACE2 affinity, including residues in the receptor-binding motif (RBM) that enhance high-affinity interactions, rendering it an optimal reference for assessing whether HKU5 displays analogous structural characteristics linked to human infectivity and interspecies transmission.

Notwithstanding these findings, limited knowledge exists on the molecular and structural determinants that govern the interactions between HKU5 RBD and ACE2. It is essential to comprehend the interface architecture, significant energy hotspots, and evolutionary adaptations of HKU5-CoV in relation to SARS-CoV-2 and MERS-CoV to assess zoonotic risk and guide treatment strategies [12,13]. Thus, this study offers comprehensive mechanistic insights into the recognition of ACE2 by HKU5 RBD by the integration of structural modeling, molecular dynamics, alanine-scanning mutagenesis, and computational peptide design. These findings are significant for cross-species transmission and receptor-targeted antiviral therapies.

This study aims to expand upon these findings by investigating rational peptide-based therapeutics and the biological factors that influence HKU5 RBD-ACE2 recognition. Initially, this study analyzed the structures of the spike proteins from HKU5 and SARS-CoV-2 in their interactions with human ACE2. The study employed all-atom molecular dynamics (MD) simulations lasting 300 nanoseconds (ns) to examine features such as interface stability and conformational dynamics. Continuous ACE2 interface residues were selected as a candidate peptide for inhibition studies. A library including 380 mutants was designed by applying systematic single-site saturation mutagenesis to a 20-mer peptide, where “mer” denotes the number of amino acid residues. Further, the top four mutants, which exhibited enhanced binding, were subjected to 300-nanosecond (ns) molecular dynamics simulations to evaluate stability and interfacial interactions. Quantum chemistry computations were employed to rapidly assess the electronic energies and border molecular orbitals (HOMO/LUMO) of the peptides to gain insights at the electronic level. Finally, the optimum peptide–HKU5 complex underwent hybrid Quantum Mechanics/Molecular Mechanics (QM/MM) single-point calculations to assess the electronic interactions between the peptide and protein. This enabled a more comprehensive mechanistic comprehension of the peptide’s impact on the binding of HKU5 to ACE2.

This study presents a methodology for systematic peptide design aimed at developing zoonotic coronaviruses and elucidates the interface factors of HKU5-ACE2 recognition. This is accomplished by a multi-scale computational framework that incorporates structural, dynamic, energetic, and quantum-level evaluations.

## 2. Methodology

### 2.1. Structure Modeling

The structure of the HKU5 receptor-binding domain (RBD) bound to human ACE2 was retrieved using the RCSB Protein Data Bank entry with PDB ID: 9JJ6 [14]. This study incorporated the structure of the SARS-CoV-2 RBD bound to ACE2 (PDB ID: 9ELE) to facilitate a comparison of the binding interactions between the two viral strains. The spike HKU5 showed some missing residues; thus, this structure was modeled using the SwissModel tool (https://swissmodel.expasy.org/) [15]. Further, the HKU5 and SARS-CoV-2 receptor-binding domains (RBDs) were aligned via BLASTP v.2.17.0 to identify conserved residues implicated in ACE2 recognition. The conservation of receptor-binding interfaces was assessed using structural superimposition of RBDs using PyMOL v 3.1.

### 2.2. Protein–Protein Docking

The HKU5 and SARS-CoV-2 receptor binding domains were docked onto ACE2 via HDOCK to generate the bound complexes [16]. Targeted docking was conducted by selecting the known interacting residues as parameters for docking. The best docked pose with preserved known interface connections was selected for each of the complexes. The interaction of critical receptor-binding residues was verified through visual inspection of docked complexes and compared with known crystal structures.

### 2.3. Alanine-Scanning Mutagenesis of RBD–ACE2 Interfaces

In order to identify critical residues that influence binding affinity, the study implemented computational alanine-scanning mutagenesis on HKU5 and SARS-CoV-2 RBDs that were complexed with ACE2. The study utilized mCSM-PPI2, a web-based computational tool designed to predict the effects of mutations on protein–protein interaction binding affinity [17]. This tool predicts changes in binding affinity (ΔΔG) upon single-point mutations, including alanine substitutions at interface residues. Residues with a substantial ΔΔG upon alanine substitution were identified as hotspots that are crucial for ACE2 recognition.

### 2.4. Molecular Dynamics Simulation

This study investigated the molecular dynamics underlying the interactions between the HKU5 RBD-ACE2 complex and also peptide-HKU5 complex. The Gromacs 2022.4 software [18], which is highly regarded in scientific circles for conducting such simulations, was employed in the investigation. The protein’s topology and charge were initially generated using the CHARMM36 force field [19]. The Particle Mesh Ewald (PME) approach [20] was utilized to accurately model electrostatic interactions at precise distances, which were necessary for capturing them. Next, the entire system was placed in water using the TIP3P model to mimic a natural biological environment [21]. The system’s charge was neutralized using Na^+^ and Cl^−^ ions to ensure physiologically realistic conditions. Structural energy minimization was then performed with 50,000 iterations of the steepest descent algorithm to resolve steric clashes and optimize molecular geometry. The LINCS method [22] was used to constrain the bonds to keep the system stable. The system underwent an initial 100 ps equilibration in the NVT ensemble (constant volume, particle number, and temperature) with gradual heating to 310 K. Subsequently, pressure was stabilized at 1 atm using the Parrinello–Rahman barostat in the NPT ensemble (constant temperature, particle number, and pressure). During the 300-nanosecond (ns) production run, the coordinates of the molecule structure were recorded every 10 ps. Velocity scaling [23] and Parrinello–Rahman coupling [24] were employed to maintain system stability by regulating pressure and temperature. To assess the structural stability and conformational dynamics of the system, this study utilized key analytical metrics including root mean square deviation (RMSD) and root mean square fluctuation (RMSF). These parameters provided quantitative measures of both global structural changes and local residue flexibility throughout the simulation period. Additionally, the results were examined with the use of Growdea Technologies’ visual platform “Analogue,” [25,26]. This approach allowed us to gain a deep understanding of the molecular interactions between the natural compounds and SHP2 by using advance approaches and analytical tools.

### 2.5. Interface Residue Identification and Peptide Derivation

Furthermore, the interface residues were investigated using PDBePISA (Proteins, Interfaces, Structures and Assemblies) of the final structure of HKU5-ACE2 complex [27,28]. A 20-mer continuous segment of ACE2 (residues 309–328) that is involved in HKU5 binding was selected for peptide-based investigations based on continuous interface residues. The peptide that was extracted maintained the critical hydrogen-bonding and hydrophobic contacts that were observed in the complete RBD–ACE2 interface. This peptide, which was taken as the control, was further used for designing mutants.

### 2.6. Peptide Mutagenesis and Docking

A 20-mer peptide that was derived from the ACE2 interface and interacting with HKU5 RBD was subjected to single-site saturation mutagenesis. The wild-type residue was excluded from the process of systematically mutating each residue in the peptide to all 19 other standard amino acids. This method guarantees a thorough examination of the potential substitutions for each residue. A total of 380 mutants (20 positions × 19 mutations) were generated. These mutants were generated and assessed using the FoldX tool v4.030 in accordance with stability predictions. In order to facilitate processing, the mutants were divided into four files, each of which contained 100 mutations, with the exception of the final file, which contained 80 mutations. This was necessary due to the substantial number of mutations. The objective was to assess the energetic impact of each mutation (ΔΔG) on the peptide. The top four peptides were chosen based on the highest stability of each cohort. Mutants that were energetically advantageous were identified and docked with the HKU5 RBD using HDOCK with the same protocol as mentioned in Section 2.2. Subsequently, the most optimal docked pose complexes were selected for electronic structure calculations and MD.

### 2.7. Molecular Dynamics of Peptide–RBD Complexes

The same protocol as described in Section 2.4 was used to simulate the selected peptide–RBD complexes (control and top four mutants) for 300 nanoseconds (ns) using GROMACS. The structural stability and peptide–protein interactions were evaluated through trajectory analysis, which included RMSD, RMSF, hydrogen bonding, and binding energy decomposition.

### 2.8. Quantum-Chemical Analysis (DFT)

Calculations of electronic characteristics, such as total energy, HOMO, LUMO, and the energy gap, were carried out on peptides using the HF-3c method of Density Functional Theory (DFT) [29,30]. In contrast to traditional hybrid functionals like B3LYP or M06-2X, which deliver high accuracy at a considerably higher computational expense, HF-3c is tailored for efficient electronic-structure assessment of small to medium-sized biomolecules while maintaining dependable representations of noncovalent interactions. HF-3c is particularly advantageous for the comparative screening of several peptide variations, focusing on relative energy trends and frontier orbital characteristics rather than absolute energies. The HF-3c method, a hybrid of Hartree-Fock and DFT, was selected due to its computational efficacy and precision for small to medium-sized molecules [31]. The HF-3c technique was selected for its dependable equilibrium between precision and computing efficiency in peptide-based systems, combining adjustments for dispersion, basis set superposition error, and basis set incompleteness. Prior benchmarking studies have shown that HF-3c accurately replicates the structural and energetic trends of biomolecular systems, achieving precision equivalent to advanced hybrid DFT approaches, while operating several orders of magnitude more rapidly [31,32,33,34].

The Kohn–Sham equations are used to determine the electronic energy *E* in density-functional theory (DFT), which encompasses the kinetic, interaction, exchange-correlation, and nuclear repulsion components of total energy [35]. The charge and multiplicity of each peptide were meticulously assigned to accurately portray their electrical states. Prior to processing, the peptides underwent optimization and were saved as XYZ files. In order to comprehend the stability and reactivity of the peptide, DFT calculations were conducted using the Psi4 software package to calculate the total energy, HOMO, LUMO, and energy gap.

### 2.9. QM/MM Calculations of Top Peptide

A representative conformation was selected at the final pose of the trajectory for subsequent QM/MM computations, maintaining protonation states and histidine tautomers in accordance with the MD topology. The selected frame and its corresponding topology data were exported in PDB/GRO format for further analysis. The system was partitioned into two segments: one for quantum mechanics (QM) and the other for molecular mechanics (MM) [36]. The peptide and protein side chains near the binding interface were included in the QM area (residues 309–328). Link hydrogens were employed to cap covalent bonds that traversed the QM/MM boundary. Electrostatic embedding, together with the protein, solvent, and ions, was entirely contained within the MM region. This partitioning strategy allows the QM wavefunction to interact with the classical MM framework, a technique commonly utilized for biomolecular systems [37].

The “mm_pc.dat” file was created, which contains MM charges obtained from the GROMACS simulation. The charges were subsequently employed in quantum mechanical computations via ORCA’s %pointcharges directive, enabling polarization of the quantum mechanical region by the classical environment, while maintaining the geometry consistent with the molecular dynamics snapshot, utilizing single-point energies without geometric optimization [38]. The B97-3c composite DFT method was employed to conduct the QM and QM/MM calculations in ORCA (v6.x) [39,40]. This method is applicable to complex systems and efficiently resolves basis-set and dispersion faults by incorporating solvent effects through CPCM (water) [41]. The B97-3c approach was used in this study, which offers the optimal balance of computational cost and accuracy in the context of peptide–protein interactions.

Energy decomposition at the peptide–protein interface was conducted by calculating three single-point QM/MM energies on the identical system:

1. Complex: The QM area includes both peptide and interacting protein side chains, whereas the MM region comprises the remainder of the system.
2. Peptide-only: The QM region comprises solely the peptide, with side chains eliminated but preserved as molecular mechanics point charges.
3. Contacts-only: The QM region comprises solely the contacting side chains, with the peptide omitted but preserved as molecular mechanics point charges.

The QM/MM interaction energy (ΔEint QM/MM) was calculated utilizing the formula:

$$\Delta E_{intQM/MM}=E_{complex}-(E_{peptide}+E_{contacts})$$

The electronic interactions between peptides and proteins in the polarized environment are isolated by this formula. The B97-3c method was employed to reduce the basis-set superposition error (BSSE) [42]. The electrostatics of interfacial regions were enhanced in comparison to gas-phase QM/MM through the application of CPCM for bulk polarization. The Barone–Cossi conductor-like scheme was employed for solvation, which is a widely acknowledged method for biomolecular solvation modeling [41]. The formal charge and spin multiplicity of the QM region were determined by summing MM charges and expected valence, and the SCF convergence criteria were tightened to assure robustness. Methods such as dampening, DIIS resets, and increased grids were employed to resolve SCF instabilities.

## 3. Results

### 3.1. HKU5 Structure Prediction

The structure of the HKU5 receptor-binding domain (RBD) bound to human ACE2 was retrieved using the PDB ID: 9JJ6; however, a few residues were found missing in the structure of HKU5, and thus, it was modeled using the same template. The missing residues Asn61–Asn63 and Gly220–Ser225 did not participate directly in the protein–protein interface but were retained because they contribute to the overall structural integrity of the protein.

**Figure 1:**
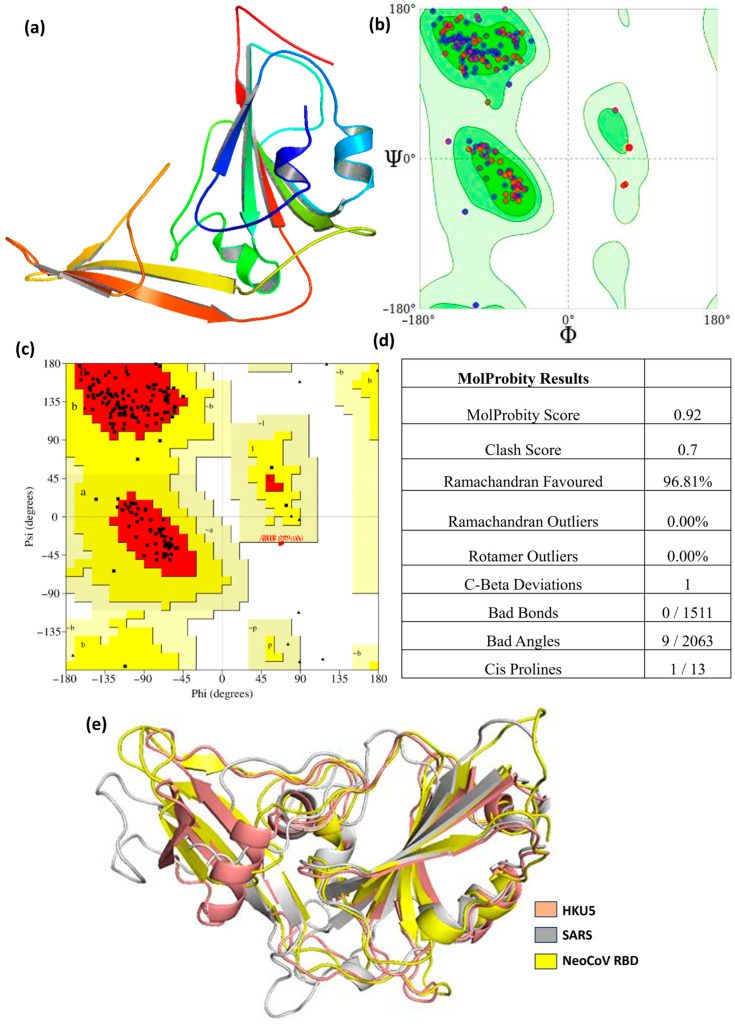
(**a**) Modeled structure of the HKU5 protein; (**b**) Ramachandran plot from Molprobity; (**c**) Ramachandran plot from PROCHECK, (**d**) Molprobity score table, and (**e**) HKU5 aligned with SARS and NeoCOV RDB.

Figure 1 depicts the predicted three-dimensional structure of the HKU5 receptor-binding domain (RBD) alongside its stereochemical confirmation by Ramachandran plot analysis. The depicted HKU5 RBD structure in Figure 1a exhibits the distinctive coronavirus RBD conformation, featuring a structured β-sheet core linked by loops and short α-helices. These secondary structural features are crucial for preserving the overall architecture necessary for receptor recognition and binding. The absent residues Asn61–Asn63 and Gly220–Ser225 were reinstated to maintain structural continuity and overall fold integrity. Significantly, these residues are situated distantly from the protein–protein interaction interface and do not directly engage in ACE2 binding; however, their presence enhances the stabilization of local loop regions and maintains the native-like structure of the RBD. The stereochemical quality of the modeled structure was assessed by Ramachandran plot analysis produced by both PROCHECK and MolProbity, as illustrated in Figure 1b,c. The Ramachandran plot illustrates the φ (phi) and ψ (psi) backbone torsion angles for all residues, facilitating the evaluation of conformational feasibility and steric integrity. MolProbity analysis produced an overall score of 0.92 and a clash score of 0.7, signifying exceptional model geometry with negligible steric conflicts shown in Figure 1d. Here, 96.81% of residues reside inside preferred regions, with 0% Ramachandran outliers, indicating a high-quality backbone conformation comparable to empirically confirmed crystal structures. The residuals occupy permissible locations, which is appropriate for homology-modeled proteins, especially in flexible loop areas. Further geometric validation indicated no erroneous bonds (0/1511), and only 9 out of 2063 bond angles exhibited minor deviations from optimal values, none of which were situated at the ACE2-binding interface. One Cβ deviation (Leu88) and a single cis-proline (Ala182–Pro183) were identified, both situated in non-interfacial and structurally flexible areas. No rotamer outliers were detected, hence reinforcing the credibility of side-chain conformations. The concordance between PROCHECK and MolProbity Ramachandran plots verifies that the rebuilt regions do not generate conformational artifacts or steric strain. The validation results indicate that the modeled HKU5 RBD structure exhibits superior stereochemical quality and structural integrity. The reconstructed missing residues do not disrupt the fold or generate interface-related distortions, thus confirming the appropriateness of this model for further docking, molecular dynamics simulations, and mutational investigations.

Further validation showed no bad bond lengths (0 out of 1511) and only a small number of bond angle deviations (9 out of 2063), none of which are located at the ACE2-binding interface or within the reconstructed loop regions, thereby minimizing their impact on protein stability or interaction analyses. A single Cβ deviation was observed at residue Leu88, which is distal to the functional interface. Only one cis-proline (Ala182–Pro183) was identified, consistent with naturally occurring cis-proline occurrences in protein structures. Collectively, these validation metrics confirm that the modeled HKU5 RBD exhibits excellent stereochemical integrity and is well-suited for molecular docking, mutational analysis, and molecular dynamics simulations performed in this study. Further, the modeled HKU5 RBD was structurally linked with the SARS-CoV and NeoCoV RBDs, both of which represent homologous merbecovirus receptor-binding domains (including the MERS-CoV lineage). Structural superposition indicated a root mean square deviation (RMSD) of 1.76 Å between HKU5 and NeoCoV RBDs, and 1.896 Å between HKU5 and SARS-CoV RBDs, as illustrated in Figure 1e. The low RMSD values imply a high degree of structural conservation among these RBDs, particularly within the core β-sheet framework, hence verifying the overall fold and loop conformations of the modeled HKU5 RBD.

**Figure 2:**
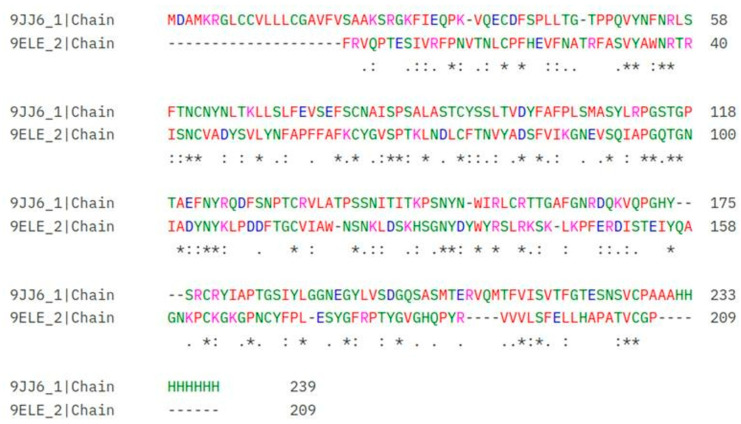
Multiple sequence alignment (MSA) of HKU5 and SARS-CoV-2 receptor-binding domains (RBDs). An asterisk (*) indicates conserved sequence (identical amino acids). A colon (:) conservative mutation (strong similarity, and (.) indicates semi-conservative mutation (weak similarity). - Gap (insertion or deletion), positions with no symbol indicate no significant similarity between the aligned residues.

The study also included the structure of the SARS-CoV-2 RBD bound to ACE2 (PDB ID: 9ELE) as a comparison to evaluate the differences in binding interactions between the two viral variants. The HKU5 and SARS-CoV-2 RBDs exhibit a moderate degree of sequence similarity in the BLASTP alignment results shown in Figure 2. The alignment encompasses 127 residues, with an identity of 29% (37/127) and 46% positivity (59/127), suggesting that the RBDs contain both conserved and divergent regions. The presence of seven gaps (5% of the sequence) indicates structural differences that may impact viral entry or receptor recognition.

The sequence alignment emphasizes numerous conserved residues, particularly those located in the middle of the sequences, including C, F, and T (positions 38–96 in the SARS-CoV-2 sequence). It is probable that these residues contribute to the RBD’s structural stability and functionality. Conversely, the alignment also reveals sequence variations that may affect the binding efficiency. For instance, in the region from YADSFVIKGNEVSQIAPGQTG (positions 79–135 in HKU5) to TVDYFAFPLSMASYLRPGSTG (positions 97–153 in SARS-CoV-2), the divergence in receptor interaction may be indicated by the significant differences in amino acid composition, which may have contributed to the differences in ACE2 binding affinity. The alignment of C-terminal residues, specifically YRSLRKS in HKU5 (positions 136–142) and IRLCRTT in SARS-CoV-2 (positions 154–160), indicated a sequence variation in a crucial region that may influence the stability and effectiveness of ACE2 recognition. The functional disparities in ACE2 recognition between the two coronaviruses may be elucidated by the differences in residues across both RBDs.

### 3.2. Docking ACE2 and Alanine Scanning Mutagenesis

The HKU5 and SARS-CoV-2 receptor-binding domains (RBDs) were bound to ACE2. By selecting known interacting residues from the ACE2-viral RBD interfaces, targeted docking was conducted, and these residues were subsequently employed as docking parameters. The optimal docked pose for each complex was selected to preserve the known interface interactions.

**Figure 3:**
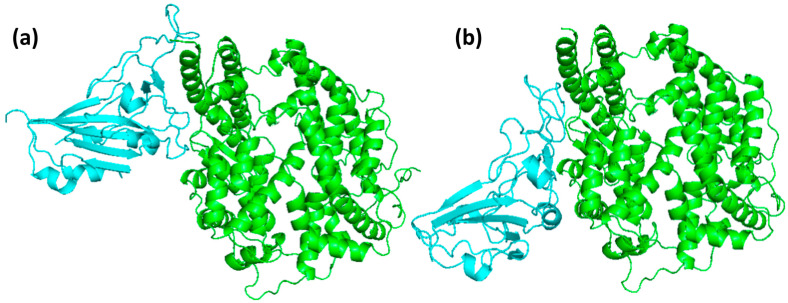
Docking pose of ACE2 (green) when bound to the (**a**) SARS-CoV-2 and (**b**) HKU5.

The HKU5 and SARS-CoV-2 RBDs docked at the same location on ACE2, as indicated by the visual analysis of the docked complexes shown in Figure 3. This alignment was in accordance with the binding interfaces that had been previously characterized. This serves as confirmation that, despite the structural and sequence differences, both viral RBDs implement analogous binding strategies to interact with ACE2. The interaction of critical receptor-binding residues in both complexes was confirmed by the visual examination of the bound poses, thereby ensuring the preservation of key residues involved in ACE2 recognition. This discovery is consistent with the crystal structures of SARS-CoV-2 and other coronaviruses, thereby emphasizing the importance of conserved receptor-binding motifs in the viral entry process. The docking scores further validated these observations; SARS-CoV-2 RBD demonstrated a docking score of −207.28, whereas HKU5 RBD demonstrated a more favorable score of −275.79. These findings suggest that both RBDs are likely to bind ACE2, with HKU5 exhibiting an even greater predicted binding propensity. Overall, the docking analysis emphasizes that the HKU5 RBD has the ability to interact with the ACE2 receptor at the canonical interface, a property that is comparable to that of SARS-CoV-2. This suggests that there is potential for ACE2-mediated entry.

**Figure 4:**
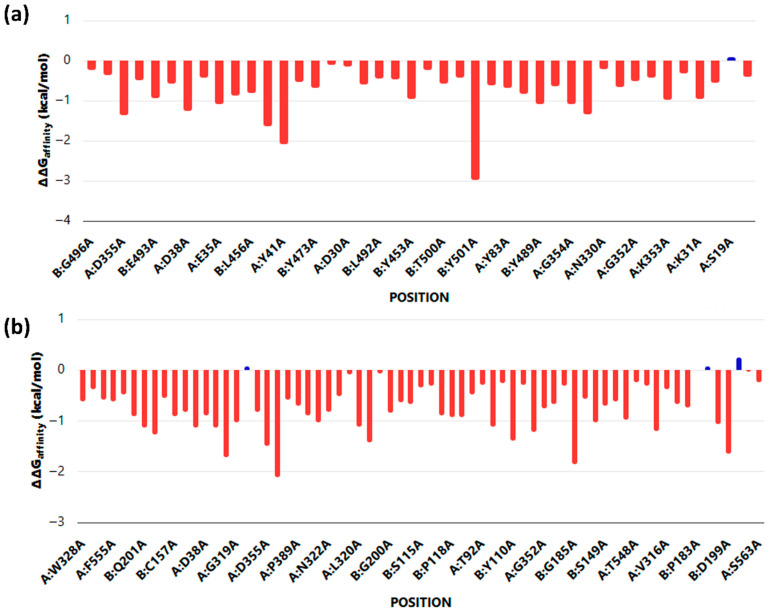
Alanine scanning mutagenesis of ACE2 when bound to (**a**) SARS and (**b**) HKU5. Red columns represent mutations predicted to decrease binding affinity (negative ΔΔG values), indicating destabilizing effects on protein–protein or protein–ligand interactions. Blue columns represent mutations predicted to increase binding affinity (positive ΔΔG values), suggesting stabilizing effects or a potential enhancement of interactions at those positions.

Alanine scanning mutagenesis identified critical hotspot residues contributing to ACE2 binding with SARS-CoV-2 and HKU5 RBDs. For the SARS-CoV-2 complex (Figure 4a, several residues exhibited substantial destabilizing effects upon alanine substitution (ΔΔG > −2.0 kcal/mol), including Tyr473, Tyr453, and Tyr500, underscoring their essential role in stabilizing the SARS–ACE2 interface. In contrast, the HKU5 complex (Figure 4b) displayed a broader distribution of moderately destabilizing mutations, with key contributions from residues such as Asp355, Tyr323, and Lys353. Notably, SARS-CoV-2 binding was dominated by a few high-impact hotspots, whereas HKU5 relied on a wider network of moderate contributors, reflecting a less optimized interface.

### 3.3. Molecular Dynamics Simulation (With ACE2 Complex)

**Figure 5:**
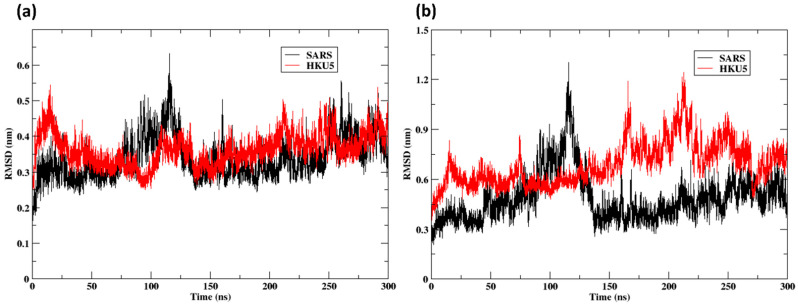
RMSD of the protein Cα atoms: (**a**) ACE2 when bound to the SARS and HKU5; (**b**) SARS and HKU5 when bound to the ACE2.

The RMSD study indicates that the SARS-CoV-2-ACE2 complex has more conformational stability than the HKU5 complex. Figure 5a illustrates diminished fluctuations (0.2–0.5 nm) in the ACE2-bound SARS-CoV-2 variant and heightened deviations (>200 ns) in the HKU5-bound variant with time. In comparison, the HKU5 RBD exhibits a variation of up to 1.2 nm, whereas the SARS-CoV-2 RBD has more stability when bound to ACE2, as indicated by lower RMSD values (0.3–0.6 nm) (Figure 5b). The findings support the idea that the binding affinity and compatibility of the SARS-CoV-2:ACE2 complex surpass those of HKU5, indicating a stronger and prolonged interaction in the former.

Despite the ability of the SARS-CoV-2 and HKU5 RBDs to bind ACE2, they engage the receptor through distinct mechanisms, as supported by the accompanying structural analyses. The SARS-CoV-2 RBD exhibits a strong affinity for the ACE2 α1 helix by fitting into a broad and constricted interface. In contrast, the HKU5 RBD engages with a small inner segment of the α1 helix of ACE2, indicating a different recognition mechanism marked by reduced or less robust stabilizing contacts.

Furthermore, recent studies indicate that SARS-CoV-2 demonstrates greater efficacy in using human ACE2 compared to specific HKU5 lineages derived from bats (e.g., HKU5-CoV-2). This aligns with the observation that the HKU5 complex had elevated RMSD fluctuations, signifying suboptimal binding interactions and increased conformational flexibility.

The conformational dynamics of ACE2 bound to SARS-CoV-2 and HKU5 RBDs were examined over a 300-nanosecond (ns) molecular dynamics trajectory (Figure S1). At the initial pose (0 ns), both SARS-CoV-2 and HKU5 RBDs occupied the canonical ACE2 binding interface, consistent with their docked configurations. By the end of the simulation (300 ns), ACE2 in complex with the SARS-CoV RBD maintained a relatively stable conformation, with only minor adjustments at the binding interface, reflecting the lower RMSD fluctuations (~0.5 nm). In contrast, the HKU5 RBD–ACE2 complex displayed more pronounced structural rearrangements, with larger deviations in the binding orientation and increased flexibility in the ACE2 interface region, consistent with higher RMSD values (~1.2 nm) observed in the trajectory. These findings suggest that while both RBDs remain bound to ACE2, the SARS-CoV-2 complex is structurally more stable, whereas HKU5 adopts a more dynamic interaction, potentially reflecting weaker but persistent binding.

**Figure 6:**
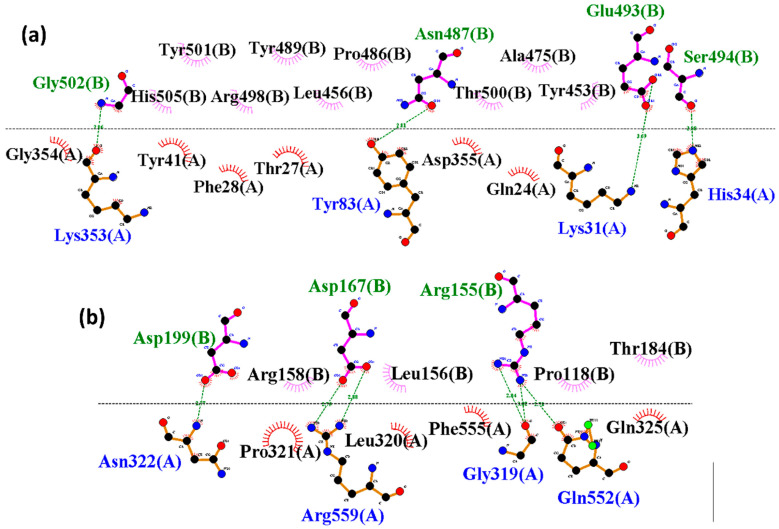
Two-dimensional interaction analysis at the final pose (300 ns) between the ACE2 with (**a**) SARS and (**b**) HKU5. Blue shows ACE2 residues, while green shows SARS-CoV-2 and HKU5 residues, respectively, with hydrogen bonds, and black with colored fans shows hydrophobic interacting residues.

The 2D interaction profiles of the SARS-CoV-2 and HKU5 RBDs bound to ACE2 at the final 300-nanosecond (ns) simulation snapshot are shown in Figure 6. In the SARS-CoV-2 RBD–ACE2 complex (Figure 6a), several well-established receptor-binding residues were preserved, including Lys31, His34, Tyr83, Asp355, and Lys353 of ACE2, forming hydrogen bonds and electrostatic contacts with key RBD residues such as Asn487, Glu493, Ser494, and Gly502. These interactions closely resemble the experimentally known SARS-CoV-2–ACE2 binding interface, explaining the relatively high stability of the complex during simulations.

In contrast, the HKU5 RBD–ACE2 complex (Figure 6b) exhibited a distinct interaction network. Here, ACE2 residues such as Asn322, Arg559, Gly319, and Gln552 engaged with HKU5 RBD residues including Asp199, Arg155, and Asp167. While these contacts contributed to maintaining binding, they involved a different subset of ACE2 interface residues compared to SARS-CoV-2. This suggests that HKU5 employs a partially shifted binding mode, relying on alternative contacts rather than the canonical hotspots observed in SARS-CoV-2.

Overall, these findings highlight that although both viruses utilize the same ACE2 binding surface, SARS-CoV-2 maintains interactions with canonical hotspot residues, leading to higher stability, whereas HKU5 exhibits a modified binding network, which may underlie its weaker binding affinity but retained binding capability.

Post-MD analyses provided further insights into the conformational dynamics of ACE2 and the viral RBDs. The RMSF profile (Figure S2a) revealed overall similar residue-wise flexibility for both complexes, but HKU5-binding induced slightly higher fluctuations at selected loop regions, indicating local structural perturbations of ACE2. The solvent accessible surface area (SASA) of ACE2 (Figure S2d) gradually decreased upon binding to SARS-CoV-2, whereas HKU5 maintained higher SASA values, suggesting looser packing and more solvent exposure. A similar pattern was observed for the viral RBDs (Figure S2e), where HKU5 exhibited persistently higher SASA relative to SARS-CoV-2, consistent with weaker interface compaction. The radius of gyration (Rg) analysis further supported these observations: ACE2 bound to SARS-CoV-2 displayed more compactness and stable Rg (2.5–2.55 nm), while HKU5 showed subtle increases in Rg over time (Figure S2f). Likewise, the HKU5 RBD exhibited greater fluctuations in Rg compared to SARS-CoV-2 (Figure S2g). Together, these results highlight that the SARS-CoV-2:ACE2 complex adopts a more compact and stable conformation, whereas HKU5 binding is associated with higher flexibility, solvent exposure, and reduced structural stability.

Supplementary Figure S2b,c display the root mean square fluctuation (RMSF) profiles of the SARS-CoV-2 and HKU5 receptor-binding domains (RBDs), respectively. The HKU5 RBD displays heightened RMSF values (>0.3 nm) at residues 68, 77–78, 80–87, 140–142, 147, 163, 179, 191–192, and 219, signifying augmented local flexibility in these areas. Significantly, none of these residues align with the rebuilt segments (Asn61–Asn63 and Gly220–Ser225), indicating that the predicted absent portions do not facilitate increased structural variations. The flexible residues are predominantly situated in loop areas distant from the ACE2-binding interface, indicative of inherent conformational mobility rather than modeling mistakes. The RMSF analysis collectively affirms the structural dependability of the modeled HKU5 RBD and verifies that the reconstruction of absent residues did not introduce artificial instability into the system.

**Figure 7:**
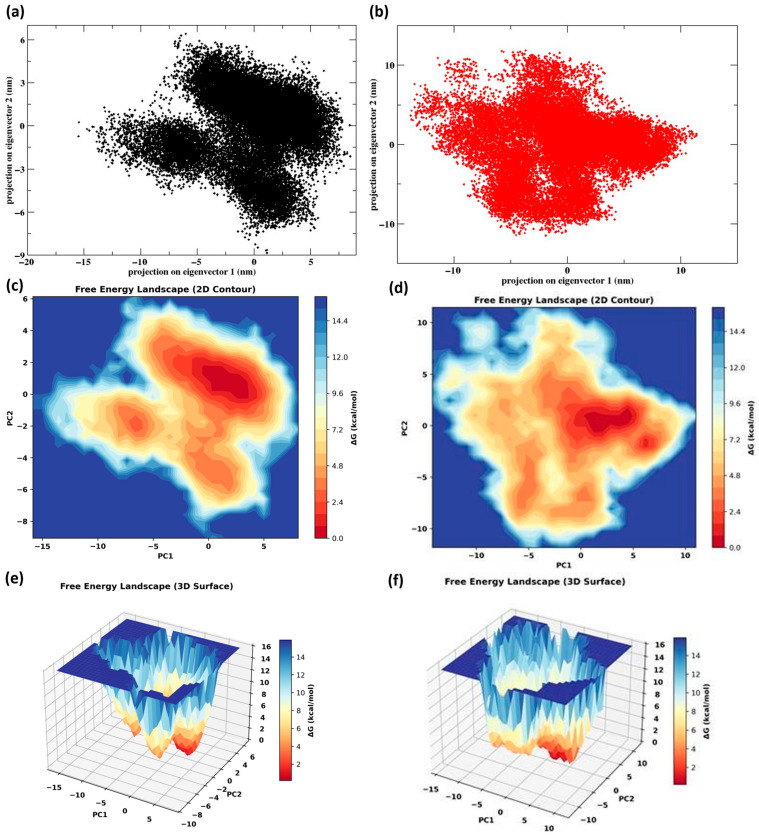
Post-MD analysis: (**a**) PCA of the protein Cα atoms ACE2 when bound to the SARS-CoV-2 and (**b**) HKU5; (**c**,**e**) FEL of ACE2 when bound to the SARS-CoV-2; (**d**,**f**) FEL of ACE2 when bound to the HKU5.

Principal component analysis (PCA) revealed distinct conformational sampling patterns for ACE2 when bound to SARS-CoV-2 and HKU5 RBDs. The SARS-CoV-2 complex (Figure 7a) occupied a relatively restricted conformational space, suggesting a stable ensemble of states, whereas the HKU5 complex (Figure 7b) explored a broader distribution, indicative of higher conformational flexibility. Free energy landscape (FEL) analysis further corroborated these findings. The SARS-CoV-2 complex (Figure 7c–e) displayed one dominant deep energy basin with a well-defined minimum, reflecting a stable and energetically favorable binding state. In contrast, the HKU5 complex (Figure 7d–f) exhibited a shallower and more dispersed energy surface with multiple minima, implying structural heterogeneity and reduced stability. Overall, these results indicate that SARS-CoV-2-binding stabilizes ACE2 into a compact, energetically favorable conformation, whereas HKU5 induces broader conformational fluctuations and less stable binding.

**Figure 8:**
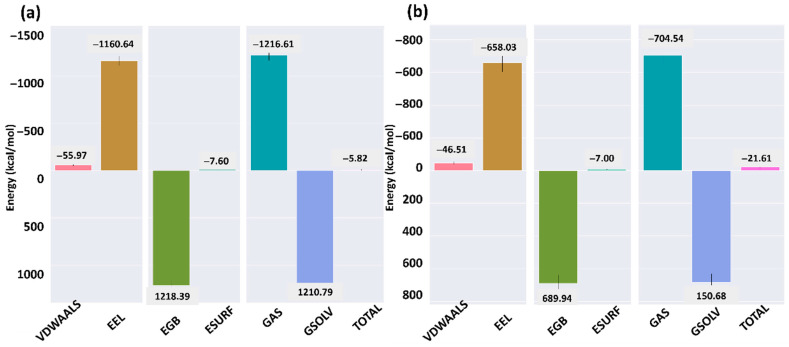
Binding free energy analysis of ACE2 when bound to the (**a**) SARS-CoV-2 and (**b**) HKU5.

The binding free energy of ACE2 in complex with the SARS-CoV-2 and HKU5 RBDs was calculated using MM/GBSA (Figure 8). For the SARS-CoV-2–ACE2 complex (Figure 8a), the total binding free energy was −5.82 kcal/mol, with major contributions from van der Waals interactions (−55.97 kcal/mol) and electrostatics (−1160.64 kcal/mol), partially offset by unfavorable solvation energies (EGB = 1218.39 kcal/mol, ESURF = −7.60 kcal/mol). In contrast, the HKU5–ACE2 complex (Figure 8b) exhibited a more favorable total binding free energy of −21.61 kcal/mol, driven by strong van der Waals (−46.51 kcal/mol) and electrostatic (−658.03 kcal/mol) components, though again offset by high polar solvation contributions (EGB = 689.94 kcal/mol, ESURF = −7.00 kcal/mol).

The comparison indicates that while both complexes are energetically stable, the HKU5 RBD–ACE2 complex displays a more favorable total free energy than the SARS-CoV-2–ACE2 complex under MM/GBSA calculations. This suggests that HKU5 has the potential to maintain ACE2 binding with comparable, if not stronger, energetic stability, despite its altered interaction network observed in the structural analysis.

Despite MM/GBSA calculations demonstrating that the HKU5–ACE2 complex possesses a more advantageous total binding free energy (−21.61 kcal/mol) compared to the SARS-CoV-2–ACE2 complex (−5.82 kcal/mol), this energetic superiority should be evaluated in conjunction with the structural evidence. Notably, although the HKU5–ACE2 complex exhibits a more favorable MM/GBSA binding free energy than the SARS-CoV-2–ACE2 complex, it also displays higher RMSD fluctuations, indicating increased conformational flexibility. This apparent contrast between energetic favorability and structural stability is further examined. MD studies revealed that HKU5 exhibits increased RMSD fluctuations and enhanced interface mobility, suggesting a more flexible and less optimized binding surface in contrast to the well-stabilized SARS-CoV-2 RBD–ACE2 interface. This apparent discrepancy illustrates an entropic compensation effect, wherein a structurally “looser” interface can yield favorable MM/GBSA ΔG due to significant electrostatic or van der Waals contributions, despite lacking the compactness and conformational stability typical of high-efficiency viral entry. Consequently, HKU5’s higher estimated ΔG does not indicate enhanced infectivity; instead, it implies that HKU5 can bind to ACE2 with adequate affinity, although its increased structural flexibility may diminish binding efficiency and conformational stability necessary for effective host–cell entrance. These findings substantiate HKU5’s zoonotic potential while concurrently emphasizing its inadequate adaptability to human ACE2 in comparison to SARS-CoV-2.

### 3.4. Interface Residue Identification and Peptide Design

To identify the critical binding interface between ACE2 and HKU5, protein–protein interface analysis was performed using PDBePISA. The analysis revealed a continuous stretch of residues spanning positions 309–328 of ACE2 (Table 1) that consistently participated in intermolecular contacts with the HKU5 RBD during binding. This segment included residues such as Lys309, Glu312, Lys313, Phe314, Phe315, and Trp328, which contribute significantly to hydrogen bonding, hydrophobic interactions, and stabilization of the ACE2–HKU5 interface. Given its continuous nature and strong interaction profile, this region was selected as the template for peptide design, with the rationale that short peptides mimicking this ACE2 stretch could competitively bind the HKU5 RBD. By capturing the essential binding determinants of ACE2, the designed peptides were expected to act as potential inhibitors, disrupting the HKU5–ACE2 interaction and thereby mitigating viral entry.

### 3.5. Peptide Mutagenesis and Docking

A 20-mer peptide derived from the ACE2 interface (residues 309–328, sequence: KEAEKFFVSVGLPNMTQGFW), where “mer” denotes the number of amino acid residues interacting with the HKU5 RBD, was subjected to single-site saturation mutagenesis. Each position in the peptide was systematically mutated to all other 19 standard amino acids (excluding the wild type), generating a total of 380 mutants (20 positions × 19 mutations). Mutants were generated and assessed using the FoldX tool, which calculated stability predictions and ΔΔG values. For computational efficiency, the mutants were divided into four cohorts (100 mutations each, with the final cohort containing 80). The stability of each of the peptides was listed in the Supplementary Sheet.

To thoroughly investigate the mutational landscape of the ACE2-derived peptide while ensuring computational feasibility, the 380 single-site saturation mutants were categorized into four cohorts. Cohorts were randomly assigned to ensure equal representation of N-terminal/C-terminal peptide residues, with no correlation to mutation type or physicochemical properties. This categorization was executed only for the purposes of workflow management and file organization, rather than being predicated on residue position, physicochemical classification, or mutation type. Each batch had roughly 100 mutations (with the final cohort consisting of 80), guaranteeing impartial representation of the complete peptide sequence across all locations. Mutational effects were assessed using FoldX by determining the alteration in folding free energy (ΔΔG) for each mutant compared to the control peptide. From each cohort, the mutant with the most favorable energy profile (lowest ΔΔG) was chosen, resulting in four leading candidates (mutant-1 to mutant-4) for further examination. This selection technique guaranteed that the final peptides embodied the most stable variations throughout the whole mutational landscape, rather than being concentrated around a singular residue location. Accordingly, the four selected mutants (mutant-1 to mutant-4) represent the most energetically favorable candidates from the full mutational space rather than artifacts of cohort partitioning. A Supplementary Table S1 has been supplied to illustrate that these candidates were not chosen arbitrarily, listing the top 20 mutants ranked by ΔΔG. The data affirm that the four chosen mutants consistently rated as the most stable and highest-affinity mutations among all the mutations, warranting their progression to docking, molecular dynamics simulations, and quantum-mechanical studies.

The most stable mutant was selected, resulting in four top candidates (mutant-1 to mutant-4), as shown in Table 2. These energetically favorable peptides were then docked with the HKU5 RBD using the HDOCK server following the same docking protocol described in Section 2.2. The best docked complexes for the control and mutants were selected for subsequent MD simulations and electronic structure calculations.

Docking results (Table 2) revealed that several mutants exhibited improved binding scores compared to the control peptide. The control peptide had a docking score of −117.44, while mutant-1 (−129.39) and mutant-3 (−129.71) demonstrated substantially enhanced docking affinities. Mutant-4 (−122.39) also outperformed the control, whereas mutant-2 (−118.66) showed modest improvement. These findings highlight the utility of mutagenesis-driven design for enhancing peptide binding against HKU5 RBD.

### 3.6. Molecular Dynamics of Peptide–RBD Complexes

**Figure 9:**
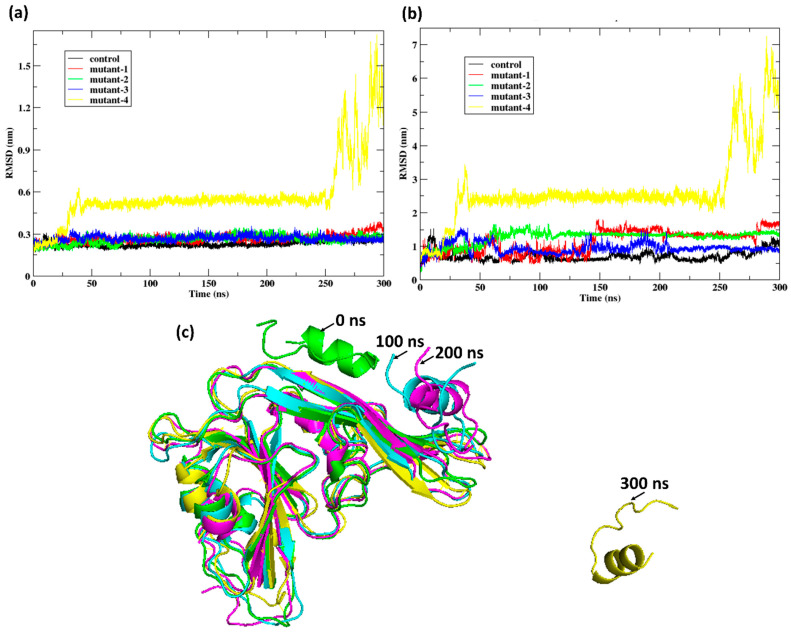
Post-MD analysis: (**a**) RMSD of the protein Cα atoms HKU5 when bound to the peptides (control, mutant-1, mutant-2, mutant-3, and mutant-4) and (**b**) RMSD of the peptides (control, mutant-1, mutant-2, mutant-3, and mutant-4) when bound to HKU5. (**c**) Conformation of the mutant-4 when bound to HKU5 during the 300-nanosecond (ns) trajectory.

The RMSD profiles revealed distinct stability patterns among the peptide variants bound to HKU5. For the receptor (Figure 9a), the control and mutants 1–3 maintained relatively stable conformations with RMSD fluctuations below 0.6 nm throughout the 300 nanoseconds (ns) trajectory. In contrast, mutant-4 exhibited a sharp increase in RMSD after 250 ns, reaching values above 1.5 nm, indicative of substantial structural deviation and instability. A similar trend was observed for the peptides (Figure 9b), where the control and mutants 1–3 remained stably bound (RMSD < 2 nm), whereas mutant-4 displayed a dramatic rise beyond 6 nm, reflecting dissociation tendencies and conformational destabilization. Structural snapshots (Figure 9c) confirmed these observations: mutant-4 progressively deviated from its bound state, culminating in unfolding and loss of binding interactions at 300 ns. These results indicate that mutants 1–3 retain favorable binding to HKU5, while mutant-4 fails to maintain structural integrity, highlighting its reduced suitability as a potential inhibitory peptide.

Following the instability of mutant-4 observed in earlier simulations, subsequent analyses were not performed for mutant-4. The RMSD plots of HKU5 (Figure S3a) indicated that the receptor remained relatively stable across all complexes, with fluctuations maintained below 0.4 nm throughout the 300-nanosecond (ns) trajectory. For the peptides (Figure S3b), the control showed the lowest deviations (0.5–1.0 nm), whereas mutant-1 and mutant-2 exhibited moderately higher fluctuations (up to 1.5 nm), and mutant-3 displayed the largest conformational variability, occasionally exceeding 2.0 nm. These results suggest that while HKU5 itself preserves structural stability upon binding, the peptides differ in their conformational robustness, with the control maintaining the most stable bound conformation, followed by mutant-1 and mutant-2, and finally mutant-3, which demonstrates reduced stability. The removal of mutant-4 was thus justified, as it failed to sustain stable binding in long-timescale simulations.

The post-MD analyses provided additional insights into the conformational behavior of HKU5 when bound to the control and mutant peptides. The RMSF profile (Figure S4a) showed that residue-level fluctuations of HKU5 remained largely consistent across all complexes, with only minor increases in flexibility observed in loop regions for mutant-1 and mutant-3. The radius of gyration (Rg) analysis of HKU5 (Figure S4b) indicated that the receptor retained overall compactness (1.75–1.85 nm), although mutant-1 complexes displayed slightly elevated Rg values, suggesting reduced compactness relative to the control and other mutants. Similarly, the Rg of the peptides (Figure S4c) highlighted differences in conformational stability: the control and mutant-2 maintained more compact states, whereas mutant-1 and mutant-3 exhibited higher fluctuations, indicating a tendency toward less stable conformations. Complementary SASA profiles (Figure S4) confirmed these trends, with mutant-1 consistently showing higher solvent exposure compared to the control and other mutants. The findings demonstrate that while HKU5 itself remains structurally stable across complexes, the peptides differ in their conformational robustness, with mutant-2 displaying the most favorable stability, followed by the control, whereas mutant-1 and mutant-3 show reduced compactness and stability.

The hydrogen bond (H-bond) analysis provided further insights into the stability of peptide binding with HKU5. The control peptide consistently maintained 3–6 H-bonds throughout the 300-nanosecond (ns) simulation (Figure S5a), reflecting strong and stable interactions. Mutant-1 (Figure S5b) displayed a similar pattern, though with slightly reduced persistence of H-bonds, maintaining 2–5 on average. Mutant-2 (Figure S5c) showed fewer stable contacts, typically fluctuating between 1 and 4 H-bonds, suggesting weaker binding affinity. Mutant-3 (Figure S5d) exhibited the highest variability, with intermittent peaks of up to 9–10 H-bonds but also long intervals with fewer than 2 bonds, indicating unstable and transient binding. Overall, the control and mutant-1 demonstrated the most consistent hydrogen-bonding interactions with HKU5, whereas mutant-2 and especially mutant-3 exhibited weaker or more fluctuating contacts, correlating with their reduced structural stability observed in RMSD, Rg, and SASA analyses.

**Figure 10:**
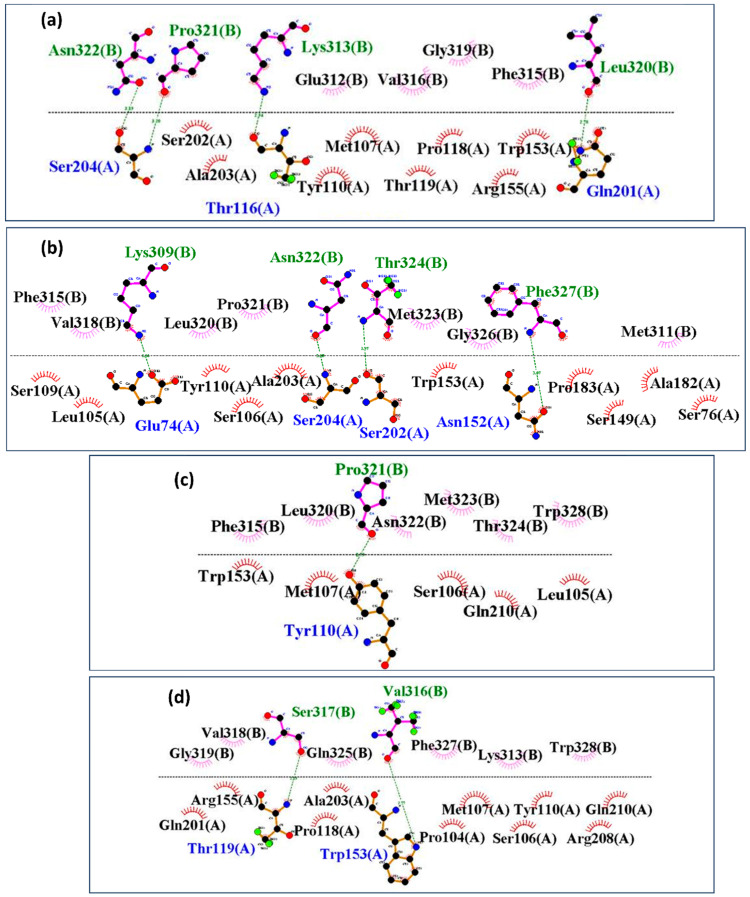
Post-MD analysis of interactions between the HKU5 when bound to (**a**) control, (**b**) mutant-1, (**c**) mutant-2, and (**d**) mutant-3. Blue shows HKU5 residues, while green shows peptide residues, respectively, with hydrogen bonds, and black with colored fans shows hydrophobic interacting residues.

Interaction mapping revealed notable differences in the binding interfaces of HKU5 with the control and mutant peptides. The control peptide (Figure 10a) established multiple stable hydrogen bonds and hydrophobic contacts, particularly involving residues Thr116, Ser204, and Gln201 from HKU5 (chain A) with Lys313, Pro321, Asn322, and Leu320 of the peptide (chain B), indicating a compact and well-stabilized interface. Mutant-1 (Figure 10b) formed additional hydrogen bonds compared to the control, involving Glu74, Ser202, Ser204, and Asn152, which contributed to enhanced stability and stronger packing within the binding pocket. Mutant-2 (Figure 10c), however, displayed fewer stabilizing contacts, with interactions limited primarily to Tyr110, suggesting a weakened binding interface. Mutant-3 (Figure 10d) exhibited altered contact patterns, including unique stabilizing bonds with Thr119 and Trp153, though with fewer consistent hydrogen bonds than mutant-1. Therefore, these results suggest that the control and mutant-1 establish the strongest and most stable interactions with HKU5, whereas mutant-2 shows the weakest binding, and mutant-3 adopts an intermediate interaction profile with altered contact geometry.

Principal component analysis (PCA) revealed the extent of conformational sampling of HKU5 in complex with the control and mutant peptides. The control complex (Supplementary Figure S6a) explored a relatively compact conformational space, reflecting stable structural dynamics. Mutant-1 (Figure S6b) exhibited broader sampling with two major clusters, suggesting increased conformational flexibility compared to the control. Mutant-2 (Figure S6c) showed the widest distribution, spanning multiple distinct clusters, indicative of significant structural rearrangements and reduced stability. Mutant-3 (Figure S6d) occupied an intermediate space, with a distribution broader than the control but more constrained than mutant-2, highlighting moderate conformational flexibility. These results confirm that the control complex is the most stable, mutant-1 displays moderate flexibility, mutant-3 occupies an intermediate state, and mutant-2 shows the highest structural variability, consistent with its weaker binding interactions observed in RMSD, Rg, SASA, and H-bond analyses.

The MM/GBSA binding free energy analysis further quantified the interaction strength of HKU5 with the control and mutant peptides. The control complex exhibited a total binding free energy of −26.44 kcal/mol (Table 3), reflecting favorable interactions. Mutant-1 (Table 3) demonstrated the strongest binding with −37.83 kcal/mol, supported by highly favorable electrostatic (−130.84 kcal/mol) and van der Waals (−57.67 kcal/mol) contributions, indicating improved stability relative to the control. Mutant-2 (Table 3) showed moderately favorable binding (−29.98 kcal/mol), comparable to the control but with weaker electrostatic stabilization. Mutant-3 (Table 3) had a binding energy of −34.95 kcal/mol, stronger than the control and mutant-2, though slightly weaker than mutant-1. Across all systems, van der Waals and electrostatic terms provided the major stabilizing contributions, while polar solvation energies opposed binding. Taken together, these results confirm that mutant-1 forms the most energetically favorable complex with HKU5, followed by mutant-3 and mutant-2, with the control peptide serving as the reference.

**Figure 11:**
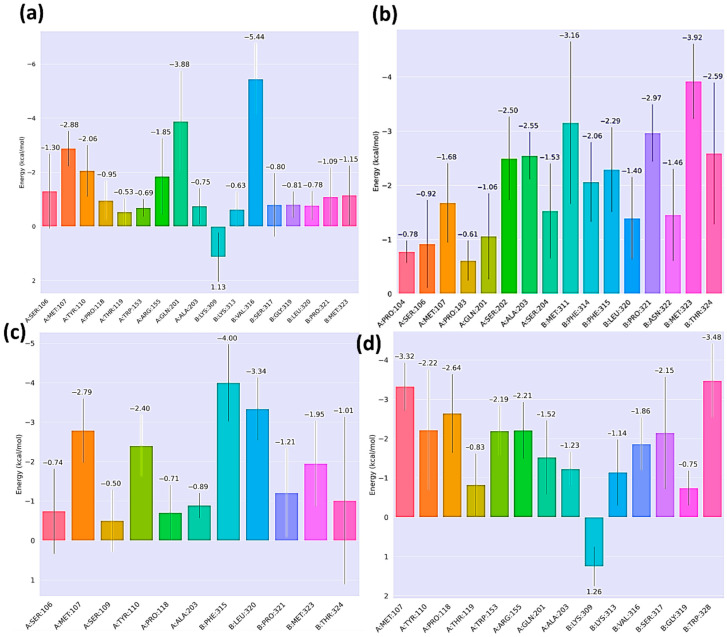
Post-MD analysis: per-residue decomposition HKU5 when bound to (**a**) control, (**b**) mutant-1, (**c**) mutant-2, and (**d**) mutant-3.

Per-residue free energy decomposition provided detailed insights into the key contributors driving HKU5–peptide binding. It was observed that the residues Met107, Ala203, and Pro118 of the HKU5 contributed to the binding free energy for all the peptides. For the control complex (Figure 11a), residue Val316 (−5.44 kcal/mol) from the peptide contributed prominently, stabilizing the peptide interface. Mutant-1 (Figure 11b) exhibited enhanced stabilization from residues Met323 (−3.92 kcal/mol), Thr324 (−2.59 kcal/mol), and Met311 (−3.16 kcal/mol), consistent with the improved total binding free energy observed in MM/GBSA. Mutant-2 (Figure 11c) showed comparatively weaker contributions, with only Phe315 (−4 kcal/mol) and Leu320 (−3.34 kcal/mol) providing modest stabilization, while several residues contributed unfavorably, reflecting its reduced binding affinity. Mutant-3 (Figure 11d) displayed moderate stabilization, with notable contributions from Trp328 (−3.48 kcal/mol) and Ser317 (−2.15 kcal/mol), though less consistent than mutant-1. Overall, these results highlight that mutant-1 achieves the strongest and most favorable residue-specific interactions with HKU5.

### 3.7. Quantum-Chemical Analysis (DFT)

Quantum-chemical (DFT) calculations were conducted on the optimized geometries of the wild-type (control) and mutants to further investigate the electronic structure of the peptide–protein complexes. The total electronic energy (E, Hartree), the frontier orbital energies (HOMO, LUMO), and the corresponding HOMO–LUMO energy gap (eV) were computed. These parameters provide a measure of electronic stability and reactivity, as illustrated in Table 4.

Mutant-1 exhibited the highest overall stabilization at the electronic level, as evidenced by its negative total energy (−8452.42 Eh) among the systems. It is also notable that mutant-1 exhibited a relatively larger HOMO–LUMO gap (0.216 eV) in comparison to the control (0.061 eV) and other mutants. This suggests that mutant-1 exhibited improved electronic stability and a reduced propensity for charge transfer. In contrast, mutant-3 and mutant-2 exhibited narrower gaps (0.078 and 0.141 eV, respectively), which is consistent with an increase in electronic plasticity and reactivity. In comparison to the mutant variants, the control system demonstrated the smallest gap (0.061 eV), which underscored its inferior electronic stability.

The DFT analysis taken together suggests that mutations have the potential to significantly alter the frontier orbital landscape. Mutant-1 is the most stable mutant, while mutant-3 is relatively less stable, despite its favorable QM/MM binding contributions. Mutant-1 is a plausible candidate with enhanced electronic robustness and binding stability, as these quantum-level descriptors provide complementary evidence to the interaction energy analysis.

### 3.8. QM/MM Calculations of Top Peptide

QM/MM single-point energy calculations were conducted on the final MD snapshots of the top peptide candidates to assess the energetic basis of peptide–HKU5 binding at the quantum mechanical level. The analysis concentrated on Peptide 1 (ACE2 residues 309–328), which had demonstrated promising stability during MD, and its designed variants (mutant-1 and mutant-3). For the QM/MM analysis, representative final conformations were extracted from 300-nanosecond (ns) all-atom MD trajectories.

For the QM/MM calculations, the system was partitioned into a quantum mechanical (QM) region and a molecular mechanical (MM) environment. The QM region consisted of the full peptide and key HKU5 RBD interface residues that maintained persistent interactions throughout the equilibrated molecular dynamics trajectory. Residues were selected based on their sustained hydrogen bonding, electrostatic interactions, or close spatial proximity (≤3–4 Å) to the peptide during the simulation. Accordingly, the QM region included Ser109, Leu105, Glu74, Tyr110, Ser106, Ala203, Ser204, Ser202, Trp153, Asn152, Pro183, Ser149, Ala182, and Ser76. The MM region represented the remaining HKU5 protein, solvent, and ions as fixed point charges to facilitate electrostatic embedding. This enabled the QM density of the peptide–residue cluster to polarize in response to the broader protein–solvent environment, thereby more accurately depicting interfacial electrostatics than gas-phase models.

Three distinct QM/MM single-point energies were calculated for each peptide mutant: (i) the complete complex (peptide + contact residues), (ii) the isolated peptide, and (iii) the isolated contact residues. Subsequently, the relation was employed to determine the interaction energy (ΔE_interaction):

$$\Delta E=E_{complex}-(E_{peptide}+E_{contacts})$$

The stabilizing effect of peptide–protein electronic interactions within the polarized MM environment is isolated by this scheme.

The results (Table 5 and Supplementary Figure S7) demonstrated a significant distinction between mutant-1 and mutant-3. Mutant-1 exhibited an interaction energy of −170.47 Hartree (−106,970 kcal/mol) that was exceedingly favorable. This robust stabilization is indicative of the extensive hydrogen-bonding and electrostatic network that has been established between the peptide and HKU5 interface residues. This network is particularly significant, as it involves Lys309, Glu312, Lys313, Phe315, and Trp328 of the peptide, as well as Glu74, Tyr110, and Trp153 of HKU5. Mutant-1 is a complex that is firmly stabilized, as evidenced by the persistence of these interactions during MD and their energetic reinforcement in the QM/MM analysis.

In contrast, mutant-3 generated an interaction energy of −2.67 Hartree (−1676 kcal/mol). In this instance, the complex was reliant on weakened van der Waals contacts as a result of the transient or loss of several peptide–residue hydrogen bonds during the MD trajectory. In comparison to mutant-1, the stabilization energy is significantly reduced due to the absence of stable anchoring interactions with critical HKU5 residues, including Ser109 and Tyr110.

The relative trend between mutants is robust, despite the fact that the interaction energies have large absolute values as a result of the incorporation of an extended QM region. Mutant-1 consistently exhibited stronger electronic stabilization than mutant-3, which was consistent with its superior structural stability in MD (lower RMSD and closer binding at the interface). The utility of QM/MM calculations as a refinement step beyond classical force-field approaches is underscored by these results, which facilitate a more nuanced evaluation of peptide candidates.

In general, the QM/MM analysis emphasizes mutant-1 as the most promising peptide binder to HKU5 as a result of its favorable electronic interactions and the stabilization of critical ACE2-mimicking residues within the viral binding pocket.

## 4. Discussion

This study’s multiscale computational analysis offers structural and mechanistic insights that enhance comprehension of possible zoonotic spillover and host adaptability of the bat-derived HKU5 coronavirus. The analysis of HKU5–ACE2 and SARS–ACE2 complexes demonstrated that HKU5 interacts with human ACE2 with much higher expected affinity (ΔG_total = −21.61 kcal/mol for HKU5 compared to −5.82 kcal/mol for SARS-CoV-2) and displays enhanced interfacial flexibility (RMSD reaching around 1.2 nm). High binding affinity coupled with conformational plasticity is a hallmark of developing zoonotic viruses adept at host adaptation, as evidenced in SARS-CoV-2 and other coronaviruses that attained human ACE2 use via minimal spike alterations [43].

The alanine-scanning findings emphasize this evolutionary assertion: particular interface residues in the HKU5 RBD were identified as energetic “hotspots,” and their mutation to alanine markedly reduced binding affinity. This reflects previous discoveries in SARS-CoV-2, indicating that only a limited number of RBD alterations facilitated ACE2 compatibility [44].

Moreover, our peptide-based inhibitory strategy utilizes this understanding. We produced 380 single-site mutations from a 20-mer ACE2-derived peptide and identified four top candidates. Docking and molecular dynamics simulations demonstrated enhanced binding for mutant-1, mutant-3, and mutant-4 compared to the control, with docking scores of −129.39, −129.71, and −122.39 versus −117.44 for the control. The DFT electronic studies (HOMO–LUMO gaps) and QM/MM interaction energy (−170.47 Hartree for mutant-1) indicate improved structural and electronic compatibility with HKU5 RBD. Biologically, this indicates that the peptide may successfully compete with ACE2 for RBD binding, potentially obstructing viral entry—a therapeutic notion pertinent to zoonotic prevention.

By integrating structural adaptability, binding energetics, and peptide competition into a unified framework, our research indicates that HKU5 is not only evolutionarily prepared for human ACE2 engagement but also susceptible to targeted suppression [45,46]. This twin insight—host-adaptation readiness and treatment vulnerability—underscores the pressing necessity for surveillance of HKU5-like viruses and the development of receptor-targeted inhibitors. Our findings underscore that minor structural alterations can yield significant epidemiological effects, mirroring the wider biology of the coronavirus, where fast changes in the spike protein enhance transmissibility and facilitate cross-species transmission.

The stability patterns noted in the wild-type and synthetic peptide–HKU5 complexes provide significant biological insights into the potential translation of computational energetics into viral receptor interaction and suppression. Peptide variations displaying diminished binding free energies (MM/GBSA), decreased structural fluctuations during molecular dynamics, and narrower HOMO–LUMO gaps exhibited improved molecular stability at the HKU5 RBD binding interface. These stabilizing interactions signify enhanced and more enduring binding, which is especially pertinent for competitive inhibition techniques, as stable interface creation is frequently necessary to obstruct native ACE2 interaction with viral spike proteins [47,48,49]. In contrast, the heightened conformational plasticity noted in the HKU5–ACE2 complex relative to SARS-CoV-2 corresponds with earlier research indicating that receptor-binding flexibility enhances cross-species adaptability and fosters zoonotic potential among lineage C merbecoviruses [6,8]. This adaptability is thought to facilitate tolerance of mutations at critical interface residues, allowing for affinity modulation during host transitions. Consequently, in biological terms, computational stability in this situation may act as a surrogate for viral transmissibility (when examining natural RBD–ACE2 complexes) or inhibitory potential (when assessing therapeutic peptides). These findings highlight the dual significance of stability—either as a catalyst for host adaptation in endogenous viral proteins or as an essential characteristic of efficient peptide-based entrance inhibitors.

### 4.1. Limitations

The present study offers a comprehensive computational analysis of the HKU5–ACE2 interaction and a peptide-based suppression approach, although numerous limitations must be recognized. All results—comprising binding affinities, mutational stability, conformational dynamics, and electronic properties—are obtained via in silico investigations and necessitate experimental validation. Computational free-energy estimations may inadequately account for entropic influences, solvent variability, or cellular environment. Moreover, peptide behavior in biological systems may significantly deviate from predictions owing to proteolytic breakdown, restricted bioavailability, transport obstacles, and structural flexibility in solution. This study concentrates exclusively on a singular ACE2-derived peptide scaffold; alternate approaches, like stapled peptides, macrocyclic peptides, or nanobody-based inhibitors, were not investigated and may provide enhanced stability and therapeutic efficacy. If mutant-1 exhibits proteolytic degradation in cell-based assays, future work could explore stapled peptide modifications to improve serum half-life—an approach validated for ACE2-derived inhibitors of SARS-CoV-2 [50].

### 4.2. Future Directions

Future endeavors will focus on the experimental validation of computational predictions, specifically highlighting mutant-1 as the foremost contender. The recombinant expression of the HKU5 RBD and human ACE2, followed by binding affinity assessments utilizing surface plasmon resonance (SPR) or biolayer interferometry (BLI), will be crucial for quantifying receptor interaction. Cell-based experiments utilizing ACE2-expressing systems, such as HKU5 pseudovirus entry tests in Vero or HEK293T cells, must be utilized to evaluate the capacity of mutant-1 to impede viral entry. Circular dichroism (CD) spectroscopy will be employed to validate peptide secondary structure and stability in solution, which are essential factors influencing binding efficacy. In addition to validation, expanding the design method to include combinatorial or multi-site mutagenesis, AI-assisted peptide optimization, and testing against various merbecovirus lineages may enhance inhibitor efficacy and offer more understanding of zoonotic risk and interspecies transmission.

This study provides the inaugural comprehensive analysis of the HKU5–ACE2 interface at the structural, mutational, and quantum levels, and introduces mutant-1 as a computationally validated peptide candidate with the potential to interfere with receptor binding and reduce the risk of zoonotic transmission.

## 5. Conclusions

This study elucidates the genetic mechanisms that regulate HKU5 spike RBD recognition of human ACE2 and underscores the significance for zoonotic spillover potential. This study employed an integrated computational framework to identify critical residues that influence ACE2 interaction and facilitate host adaptation. Of the 380 rationally designed mutant peptides derived from ACE2 interface residues, mutant-1 consistently proved to be the most stable and functionally advantageous variant. The results suggest that mutant-1 could function as a primary candidate peptide inhibitor that competitively obstructs HKU5 binding to ACE2. A comparative analysis with SARS-CoV-2 indicated that HKU5 demonstrates enhanced flexibility at the receptor-binding interface and a more advantageous global binding energy, hence intensifying apprehensions about its potential for cross-species transmission. This study advances comprehension of HKU5–ACE2 recognition mechanisms and delineates a clear pathway for peptide-based antiviral treatments. The designation of mutant-1 as the premier inhibitor candidate signifies significant progress and establishes the foundation for forthcoming in vitro validation and therapeutic development to address the advent of HKU5-like zoonotic coronaviruses. The insights provided herein may assist in guiding surveillance initiatives, prioritizing high-risk viral lineages, and informing the development of preemptive therapeutic strategies. Importantly, these computational findings establish a basis for subsequent experimental validation and the development of peptide-based antiviral approaches aimed at pre-spillover coronavirus threats.

## Figures and Tables

**Table 1:** Continuous interface residues of the ACE found while binding with the HKU5 using PDBePISA (proteins, interfaces, structures and assemblies); these residues were further used to design the peptide that bound to the HKU5.

Continuous Interface Residues of ACE2	
A:LYS309	A:GLY319
A:GLU310	A:LEU320
A:ALA311	A:PRO321
A:GLU312	A:ASN322
A:LYS313	A:MET323
A:PHE314	A:THR324
A:PHE315	A:GLN325
A:VAL316	A:GLY326
A:SER317	A:PHE327
A:VAL318	A:TRP328

**Table 2:** Docking scores of the top four peptides and the control.

Peptides	Sequence	Docking Scores
**Control**	KEAEKFFVSVGLPNMTQGFW	−117.44
**Mutant-1**	KEMEKFFVSVGLPNMTQGFW	−129.39
**Mutant-2**	KEAEKFFVRVGLPNMTQGFW	−118.66
**Mutant-3**	KEAEKFFVSVGLPNMRQGFW	−129.71
**Mutant-4**	KEAEKFFVSVGLPNMYQGFW	−122.39

**Table 3:** Components of binding free energy HKU5 when bound to control, mutant-1, mutant-2, and mutant-3. Energy terms are defined as follows: ΔVDWAALS—Van der Waals interaction energy between receptor and ligand. ΔEEL—Electrostatic interaction energy. ΔEGB—Polar solvation free energy calculated using the Generalized Born (GB) model. ΔESURF—Non-polar solvation energy estimated from solvent-accessible surface area (SASA). ΔGGAS—Gas-phase interaction energy (ΔVDWAALS + ΔEEL). ΔGSOLV—Total solvation free energy (ΔEGB + ΔESURF). ΔTOTAL—Total binding free energy (ΔGGAS + ΔGSOLV).

Peptides	ΔVDWAALS	ΔEEL	ΔEGB	ΔESURF	ΔGGAS	ΔGSOLV	ΔTOTAL
Control	−42.54	−52.82	74.56	−5.65	−95.36	68.92	−26.44
Mutant 1	−57.67	−130.84	159.1	−8.42	−188.51	150.68	−37.83
Mutant 2	−48.58	−63.28	89.07	−7.19	−111.85	81.88	−29.98
Mutant 3	−57.21	−51.57	82.29	−8.46	−108.78	73.83	−34.95

**Table 4:** Frontier molecular orbital (HOMO and LUMO) energies, HOMO–LUMO gap (eV), and total electronic energy (Eh) of the control peptide and designed mutants, obtained from quantum-chemical (DFT) calculations.

Peptides	E_Eh	HOMO_Eh	LUMO_Eh	GAP_eV
**Control**	−7907.94	−0.22544	−0.22318	0.061423
**Mutant-1**	−8452.42	−0.23034	−0.22238	0.21641
**Mutant-2**	−8180.18	−0.18229	−0.17711	0.140794
**Mutant-3**	−8143.32	−0.16373	−0.16085	0.078608

**Table 5:** QM/MM single-point energy calculations of peptide–HKU5 complexes. Interaction energies (ΔE_interaction) were obtained by subtracting the isolated peptide and contact residue energies from the full complex energy.

Mutant	System	Energy (Hartree)	Energy (kcal/mol)	ΔE_Interaction (Hartree)	ΔE_Interaction (kcal/mol)
**Mutant-1**	Complex	−6146.0239	−3,856,688	−170.47	−106,970
	Peptide	−5605.1832	−3,517,306		
	Contacts	−370.3717	−232,412		
**Mutant-3**	Complex	−13,351.8537	−8,378,415	−2.67	−1676
	Peptide	−8367.7670	−5,250,853		
	Contacts	−4981.4136	−3,125,884		

## Data Availability

Data can be requested from the corresponding author upon reasonable request.

## Supplementary Materials

The following supporting information can be downloaded at: https://www.mdpi.com/article/10.3390/ani16020237/s1, Figure S1: Conformational analysis of the ACE2 bound to the (a,b) SARS and (c,d) HKU5 at initial pose (0 ns) and final pose (300 ns); Figure S2: Post-MD analysis: (a) RMSF of the protein Cα atoms ACE2 when bound to the SARS and HKU5; (b) RMSF of SARS; (c) RMSF of HKU5; (d) SASA of ACE2 when bound to the SARS and HKU5; (e) SASA of SARS and HKU5 when bound to the ACE2; (f) SASA of ACE2 when bound to the SARS and HKU5; (g) SASA of SARS and HKU5 when bound to the ACE2. Figure S3: Post-MD analysis: (a) RMSD of the protein Cα atoms HKU5 when bound to the peptides (control, mutant-1, mutant-2, mutant-3) and (b) RMSD of the peptides (control, mutant-1, mutant-2, mutant-3) when bound to HKU5. Figure S4: Post-MD analysis: (a) RMSF of the protein Cα atoms HKU5 when bound to the peptides (control, mutant-1, mutant-2, mutant-3); (b) Rg of HKU5 when bound to the peptides (control, mutant-1, mutant-2, mutant-3); (c) SASA of the peptides (control, mutant-1, mutant-2, mutant-3) when bound to HKU5. Figure S5: Post-MD analysis: number of hydrogen bonds between the HKU5 when bond to (a) control, (b) mutant-1, (c) mutant-2, and (d) mutant-3. Figure S6: Post-MD analysis: (a) PCA of the protein Cα atoms HKU5 when bound to (a) control, (b) mutant-1, (c) mutant-2, and (d) mutant-3. Figure S7:QM/MM single-point energy calculations: (a) mutant-1 and (b) mutant-3. Table S1: Folding free energy (ΔΔG) of the top 20 mutant peptides; Supplementary Sheet.
