# Supplementary material for: Structural Insights into the Receptor-Binding Domain of Bat Coronavirus HKU5-CoV-2: Implications for Zoonotic Transmission via ACE2
Source: Animals (Basel). 2026 Jan 13;16(2):237. doi: 10.3390/ani16020237 (PMC12838366; doi:10.3390/ani16020237)
Supplement: Supplementary file 1 [file animals-16-00237-s001.zip › Supplementary.pdf]

Supplementary Table

Table S1. Folding free energy (ΔΔG) of the top 20 mutant peptides

Mutants	stability (kcal/mol)	Cycle
peptide_ 48	-24.09929	Cycle1
peptide_ 4	-23.10923	Cycle4
peptide_ 67	-21.98898	Cycle2
peptide_ 100	-21.74215	Cycle3
peptide_ 3	-21.68122	Cycle4
peptide_ 90	-21.67614	Cycle3
peptide_ 86	-21.45037	Cycle1
peptide_ 18	-21.445692	Cycle1
peptide_ 3	-21.244133	Cycle1
peptide_ 42	-21.05122	Cycle2
peptide_ 94	-21.03756	Cycle1
peptide_ 48	-20.97761	Cycle2
peptide_ 27	-20.87953	Cycle4
peptide_ 85	-20.8444	Cycle3
peptide_ 80	-20.7815	Cycle3
peptide_ 58	-20.70444	Cycle3
peptide_ 37	-20.59817	Cycle4
peptide_ 63	-20.36921	Cycle2
peptide_ 57	-20.27641	Cycle2
peptide_ 35	-19.97061	Cycle4

Supplementary Figures

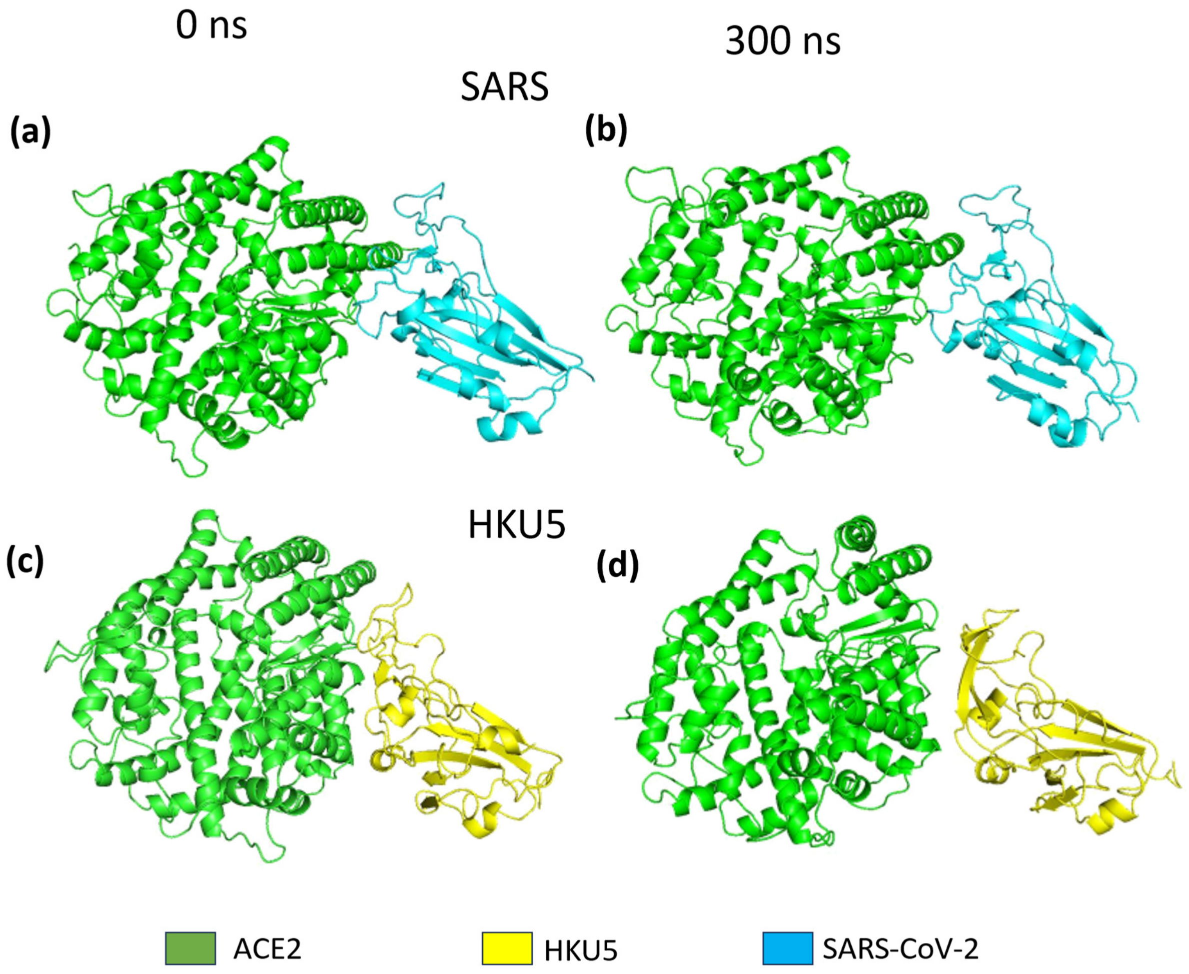

Figure S1. Conformational analysis of the ACE2 bound to the (a, b) SARS and (c, d) HKU5 at initial pose (0 ns) and final pose (300 ns)

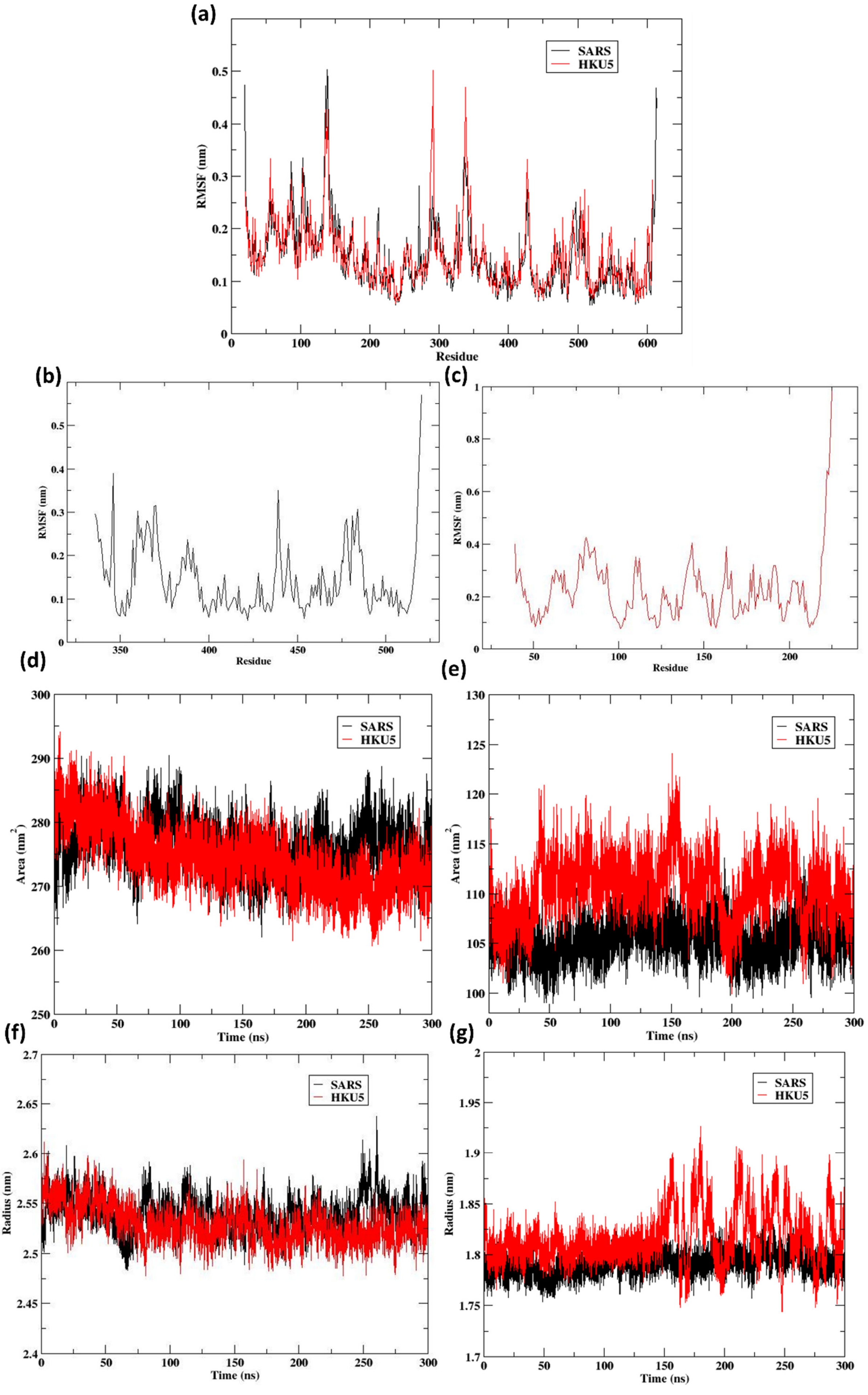

(a)
SARS
HKU5
RMSF (nm)
Residue
(b)
RMSF (nm)
Residue
(c)
RMSF (nm)
Residue
(d)
SARS
HKU5
Area (nm2)
Time (ns)
(e)
SARS
HKU5
Area (nm2)
Time (ns)
(f)
SARS
HKU5
Radius (nm)
Time (ns)
(g)
SARS
HKU5
Radius (nm)
Time (ns)

Figure S2. Post MD analysis (a) RMSF of the protein Cα atoms ACE2 when bound to the SARS and HKU5 (b) RMSF of SARS (c) RMSF of HKU5, (d) SASA of ACE2 when bound to the SARS and HKU5 (e) SASA of SARS and HKU5 when bound to the ACE2, (f) SASA of ACE2 when bound to the SARS and HKU5 (g) SASA of SARS and HKU5 when bound to the ACE2

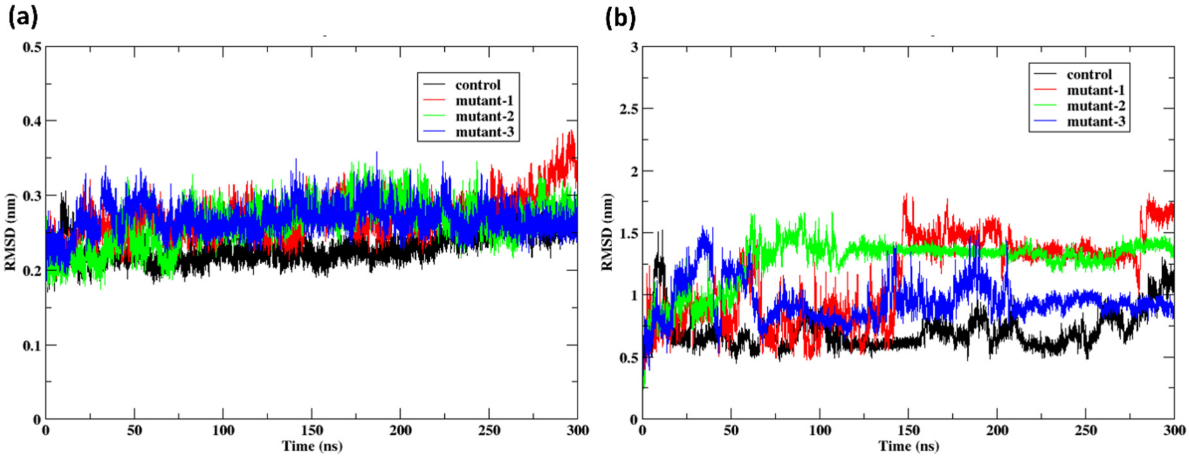

Figure S3. Post MD analysis (a) RMSD of the protein Cα atoms HKU5 when bound to the peptides (control, mutant-1, mutant-2, mutant-3) and (b) RMSD of the peptides (control, mutant-1, mutant-2, mutant-3) when bound to HKU5.

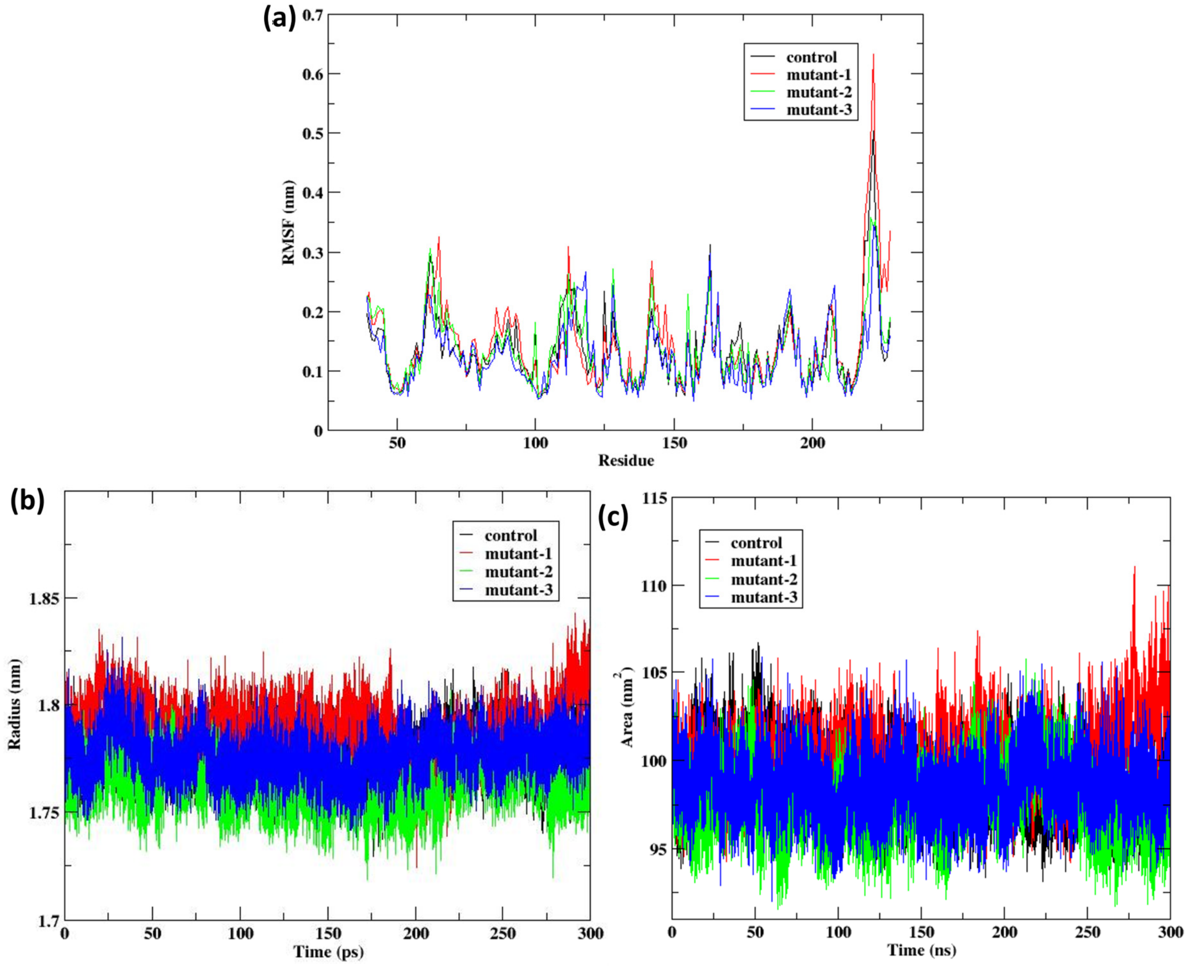

Figure S4. Post MD analysis (a) RMSF of the protein Cα atoms HKU5 when bound to the peptides (control, mutant-1, mutant-2, mutant-3) (b) Rg of HKU5 when bound to the peptides (control, mutant-1, mutant-2, mutant-3) (c) SASA of the peptides (control, mutant-1, mutant-2, mutant-3) when bound to HKU5.

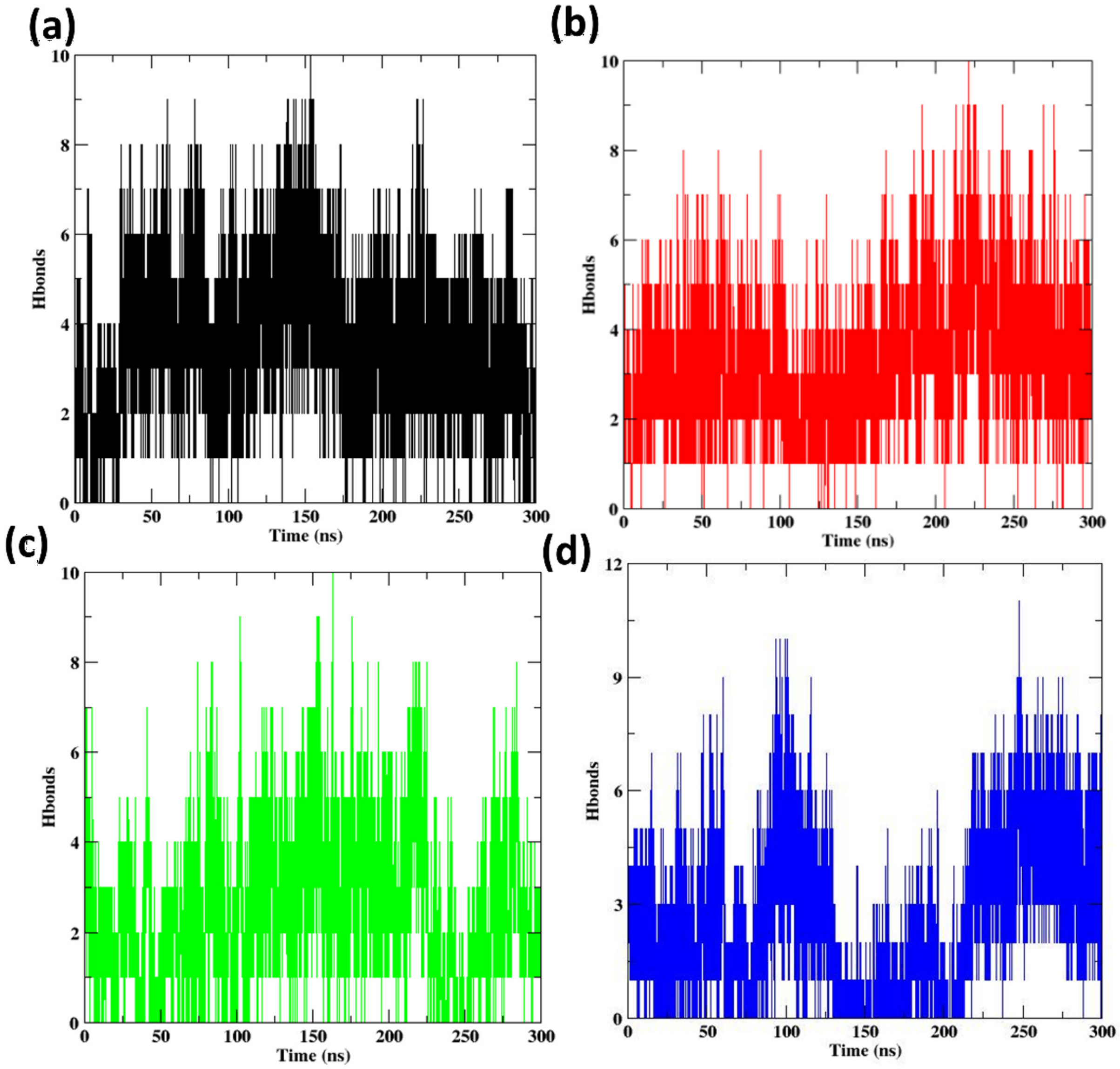

Figure S5. Post MD analysis, number of hydrogen bonds between the HKU5 when bond to (a) Control (b) Mutant-1 (c) Mutant-2 (d) Mutant-3.

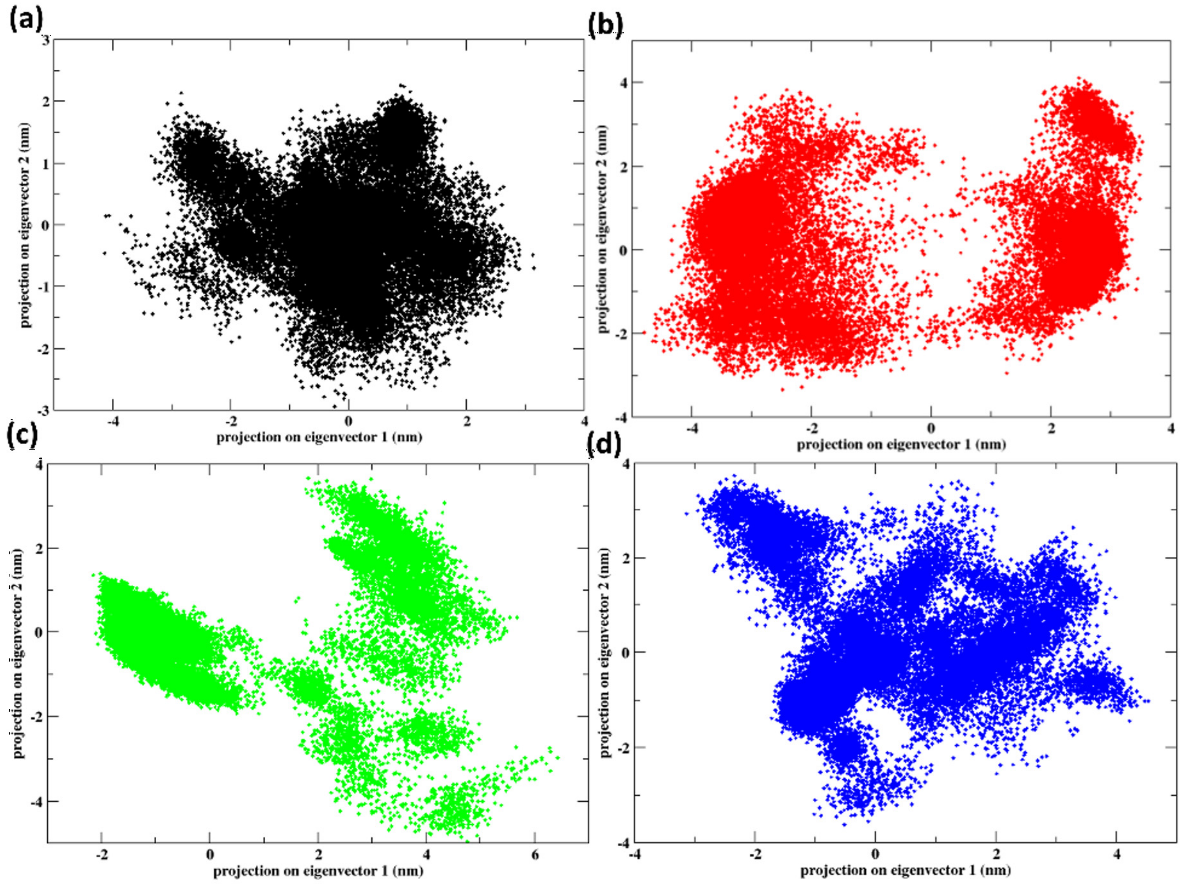

Figure S6. Post MD analysis (a) PCA of the protein Cα atoms HKU5 when bound (a) Control (b) Mutant-1 (c) Mutant-2 (d) Mutant-3

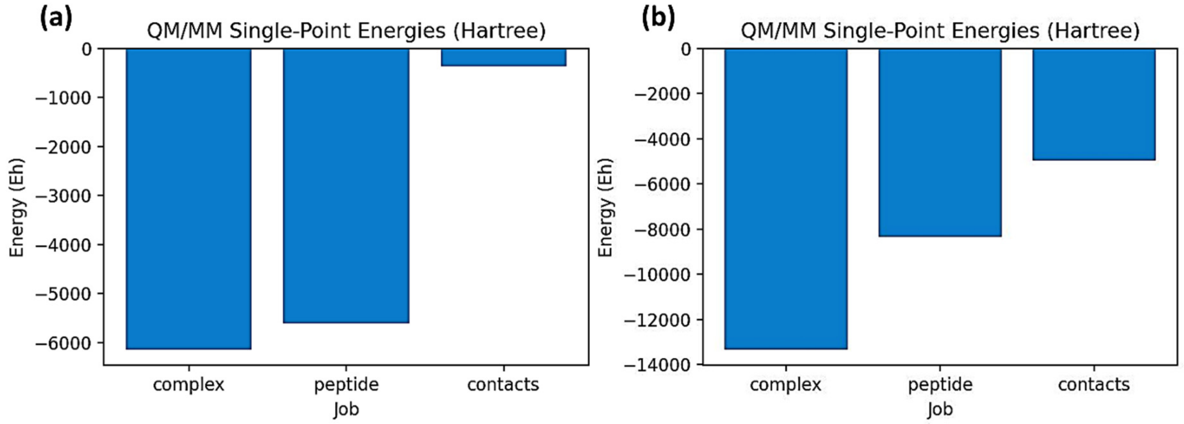

Figure S7. QM/MM single-point energy calculations (a) Mutant-1 (b) Mutant-3
